# Targeting endothelial glycolytic reprogramming by tsRNA-1599 for ocular anti-angiogenesis therapy

**DOI:** 10.7150/thno.96946

**Published:** 2024-06-01

**Authors:** Xiao-yan Han, Ling-jie Kong, Duo Li, Ming Tong, Xiu-miao Li, Chen Zhao, Qin Jiang, Biao Yan

**Affiliations:** 1 The Affiliated Eye Hospital, Nanjing Medical University, Nanjing 210000, China.; 2 Eye Institute and Department of Ophthalmology, Eye & ENT Hospital, Fudan University, Shanghai 200031, China.; 3 The Fourth School of Clinical Medicine, Nanjing Medical University, Nanjing 210000, China.; 4 Department of Ophthalmology, Shanghai General Hospital, Shanghai Jiao Tong University School of Medicine, Shanghai 200080, China.

**Keywords:** Angiogenesis, Ocular neovascular disease, Endothelial metabolism, tsRNAs, Glycolytic flux

## Abstract

***Rationale:*** Current treatments for ocular angiogenesis primarily focus on blocking the activity of vascular endothelial growth factor (VEGF), but unfavorable side effects and unsatisfactory efficacy remain issues. The identification of novel targets for anti-angiogenic treatment is still needed.

***Methods:*** We investigated the role of tsRNA-1599 in ocular angiogenesis using endothelial cells, a streptozotocin (STZ)-induced diabetic model, a laser-induced choroidal neovascularization model, and an oxygen-induced retinopathy model. CCK-8 assays, EdU assays, transwell assays, and matrigel assays were performed to assess the role of tsRNA-1599 in endothelial cells. Retinal digestion assays, Isolectin B4 (IB4) staining, and choroidal sprouting assays were conducted to evaluate the role of tsRNA-1599 in ocular angiogenesis. Transcriptomic analysis, metabolic analysis, RNA pull-down assays, and mass spectrometry were utilized to elucidate the mechanism underlying angiogenic effects mediated by tsRNA-1599.

***Results:*** tsRNA-1599 expression was up-regulated in experimental ocular angiogenesis models and endothelial cells in response to angiogenic stress. Silencing of tsRNA-1599 suppressed angiogenic effects in endothelial cells *in vitro* and inhibited pathological ocular angiogenesis *in vivo*. Mechanistically, tsRNA-1599 exhibited little effect on VEGF signaling but could cause reduced glycolysis and NAD^+^/NADH production in endothelial cells by regulating the expression of HK2 gene through interacting with YBX1, thus affecting endothelial effects.

***Conclusions:*** Targeting glycolytic reprogramming of endothelial cells by a tRNA-derived small RNA represents an exploitable therapeutic approach for ocular neovascular diseases.

## Introduction

Angiogenesis is known as the process of new blood vessel formation from pre-existing vessels. It is a highly complex process that requires intricate interactions among different vascular cells, extracellular matrix, and growth factors 1. Angiogenesis has been involved in several physiological processes, such as embryonic development, reproductive process, and wound repair. Physiological angiogenesis is critical for the balance and stabilization of the body, which can carry nutrients to the tissues and organs and remove catabolic products 2. However, pathological angiogenesis can contribute to diseased conditions, such as tumor growth, inflammatory responses, metabolic disorders, and ocular neovascular diseases 3, 4.

Angiogenesis is triggered by the imbalanced situation of increased levels of angiogenic factors and decreased levels of anti-angiogenic factors 5. Several angiogenic factors, including fibroblast growth factor (FGF), vascular endothelial growth factor (VEGF), and platelet-derived growth factor (PDGF), have been identified, which can stimulate angiogenesis through the activation of their specific tyrosine kinase receptors 6, 7. One of the most potent factors is VEGF and its receptor (VEGFR) system. Currently, it is possible to regulate VEGF-VEGFR system through anti-VEGF reagents, such as bevacizumab, ranibizumab, aflibercept, and pegaptanib. Anti-VEGF therapy has established itself as a foundational approach in the management of inflammatory diseases, tumor progression, and ocular neovascular disorders 8, 9.

Ocular angiogenesis is a major contributor to severe vision loss, occurring in a spectrum of ocular diseases such as retinopathy of prematurity (ROP), proliferative diabetic retinopathy (PDR), and wet age-related macular degeneration (wAMD) 10, 11. Current treatments for ocular angiogenesis focus on the inhibition of VEGF, a primary driver of angiogenic process. Anti-VEGF treatment can slow or stop the progression of ocular angiogenesis. However, unfavorable side effects and unsatisfactory efficiency still exist during anti-VEGF treatment 12. For example, VEGF is expressed in a variety of retinal cell types and is also known as a neurotrophic factor. Intraocular injection of anti-VEGF drug may interrupt ocular development and vascular remodeling. In addition, the potential leakage of anti-VEGF drugs into the circulation may produce systemic side effects 13. Thus, further research is still needed to identify alternative targets for anti-angiogenic treatment.

Transfer RNAs (tRNAs), transcribed by RNA polymerase III, are crucial molecules that translate the molecular information from messenger RNAs (mRNAs) into proteins, constituting approximately 15% of total RNA transcripts 14. tRNAs or pre-tRNAs can be cleaved to generate various tRNA derived small RNAs (tsRNAs), including tRNA-derived fragments (tRFs) and tRNA halves (tiRNAs) 15. These tsRNAs were previously regarded as non-functional byproducts of tRNA degradation. However, accumulating evidence has revealed that tsRNAs participate in several biological processes by regulating RNA-binding proteins, gene expression, and protein translation 16, 17. Dysregulated expression of tsRNAs has been implicated in the pathogenesis of human diseases, such as cancers, infectious diseases, and neurological disorders. 18, 19. However, the role of tsRNAs in ocular angiogenesis remains largely unclear.

In this study, we investigated the role of tsRNAs in ocular angiogenesis. We reveal that a novel tsRNA, tsRNA-1599, can inhibit pathological ocular angiogenesis. Intravitreal injection of tsRNA-1599 does not cause retinal ganglion cell (RGC) or photoreceptor injury, nor deleterious effects on mature blood vessels. Mechanistically, tsRNA-1599 exhibits no direct effects on VEGF signaling but causes glycolytic reprogramming in endothelial cells. This study reports a novel regulator of ocular angiogenesis with potential clinical application for treating ocular vascular diseases.

## Results

### tsRNA-1599 is a potential regulator of experimental neovascularization

**Figure 1:**
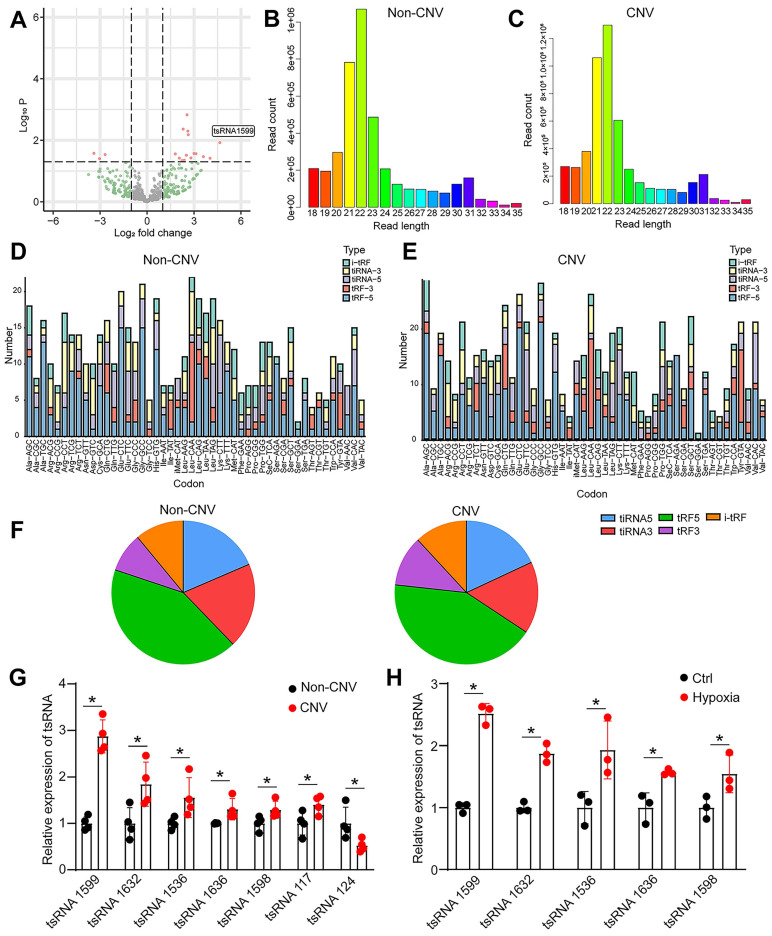
** tsRNA-1599 is a potential regulator of experimental neovascularization.** (A) Volcano plot filtering was conducted to identify differentially expressed tsRNAs in the RPE-choroid-sclera complexes between CNV group and non-CNV group. (B and C) Length distribution of tsRNAs were shown in non-CNV group (B) and CNV group (C). (D and E) Stacked plots displaying the percentage of each tsRNA sub-type sorted by the sites and length expressed in non-CNV group (D) and CNV group (E). (F) Pie charts showing the percentage of different tsRNA types in non-CNV group and CNV group. (G) qRT-PCR assays were conducted to compare the expression of the indicated tsRNAs between CNV group and non-CNV group (n = 4, *P < 0.05 vs. non-CNV, Student *t* test). (H) HUVECs were treated with CoCl_2_ (300 μmol/L) to mimic hypoxic condition or left untreated (Ctrl) for 24 h. qRT-PCR assays were conducted to detect the expression of the indicated tsRNAs in HUVECs (n = 3, *P < 0.05 vs. Ctrl, Student *t* test).

To identify and characterize tRNA-derived small RNAs (tsRNAs) involved in experimental neovascularization, four pairs of RPE-choroid-sclera complexes in CNV mice and normal mice were subjected for small RNA sequencing. According to *P* value < 0.05 and log2 Fold Change ≥ 2, 18 differentially expressed tsRNAs were identified between CNV group and non-CNV group, including 15 up-regulated tsRNAs and 3 down-regulated tsRNAs in CNV group. A volcano plot was used to display the differentially expressed tsRNAs between CNV group and non-CNV group (Figure 1A). Then, a bar graph was used to display the read length distribution of tsRNAs ranged from 15 to 35 nucleotides (Figure 1B-C). Since one type of tsRNAs can be produced from different tRNAs by the cleavages into the fragments with the identical sequences, the stacked plots displayed the percentage of each tsRNA sorted by their sites and lengths in CNV group and non-CNV group (Figure 1D-E). The percentage of different types of tsRNAs was shown (Figure 1F).

To validate the results of small RNA sequencing data, we collected the RPE-choroid-sclera complexes and detected the expression pattern of differentially expressed tsRNAs. Intriguingly, the greatest up-regulation of tsRNA-1599 expression was detected in CNV group (Figure 1G). Hypoxia is known as a critical driver of ocular neovascularization 20. HUVECs were incubated with CoCl_2_ for 24 h to mimic hypoxic condition. Hypoxic stress caused a marked increase in the detected tsRNAs (Figure 1H). Notably, the greatest up-regulation of tsRNA-1599 expression was detected in hypoxic group.

### tsRNA-1599 expression is significantly up-regulated in experimental neovascularization *in vitro* and *in vivo*

**Figure 2:**
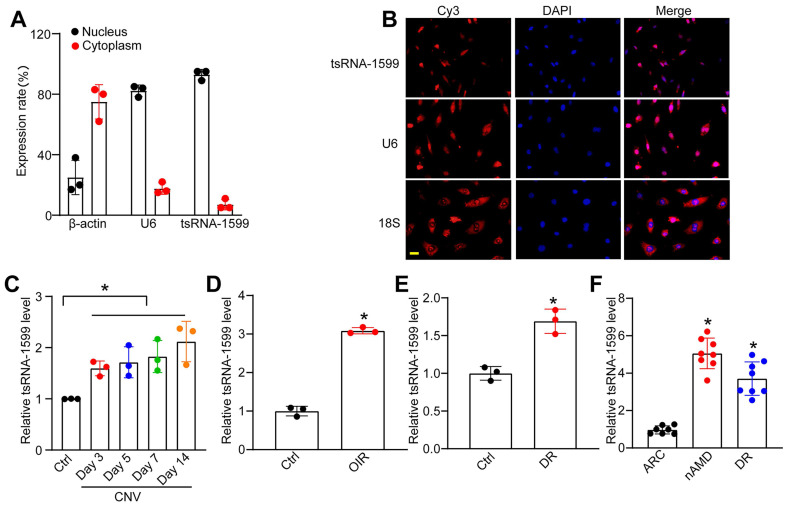
** tsRNA-1599 expression is significantly up-regulated in experimental neovascularization *in vitro* and *in vivo*.** (A) The expression levels of nucleus control transcript (U6), cytoplasm control transcript (β-actin), and tsRNA-1599 were detected by qRT-PCR assays in the nucleus and cytoplasm fraction of HUVECs (n = 3). (B) RNA-FISH assays were conducted to detect the distribution of tsRNA-1599 expression in HUVECs using Cy3-labeled probe (tsRNA-1599). Nucleus control transcript (U6) and cytoplasm control transcript (18S rRNA) was also detected. Nuclei were stained with 4ʹ, 6-diamidino-2-phenylindole (DAPI). Scale bar, 10 μm. (C) qRT-PCRs were conducted to detect the levels of tsRNA-1599 expression in the RPE-choroid-sclera complexes of C57BL/6J mice after 3, 5, 7, 14-day laser photocoagulation (n = 3, *P < 0.05 vs. Ctrl, One-way ANOVA followed by Bonferroni's post hoc test). (D) qRT-PCR assays were conducted to compare the levels of tsRNA-1599 expression in the OIR retinas and normal retinas (n = 3, *P < 0.05 vs. Ctrl, Student *t* test). (E) qRT-PCR assays were conducted to compare tsRNA-1599 expression in DR retinas and non-DR retinas (n = 3, *P < 0.05 vs. Ctrl, Student *t* test). (F) qRT-PCR assays were conducted to compare tsRNA-1599 expression in aqueous humor of nAMD patients (n = 8) or DR patients (n = 8) with age related cataract (ARC, n = 7 vs. ARC, *P < 0.05, One-way ANOVA followed by Bonferroni's post hoc test).

tsRNA-1599 is an i-tRF originating from the 3' half of mature tRNA-Tyr-GTA, with a length of 20 nucleotides. The expression of tsRNA-1599 in the nuclear and cytoplasmic fraction was assessed using qRT-PCR. The findings indicated predominant expression of tsRNA-1599 in the nucleus (Figure 2A). FISH assays also verified that tsRNA-1599 was mainly expressed in the nucleus of HUVECs (Figure 2B). Laser-induced CNV is an important experimental model that can re-create the vascular hallmarks of wAMD 21. qRT-PCR assays showed that the levels of tsRNA-1599 expression were significantly up-regulated in the RPE/choroid complexes on day 3, day 5, day 7, and day 14 following laser photocoagulation (Figure 2C). Oxygen-induced retinopathy model is a widely used model to study ischemia-driven neovascularization (NV) in the retina 22. Neonatal C57BL/6J mice were exposed to 75% oxygen from P7 to P12, and then returned to the normoxic condition for 5 days to trigger hypoxia-induced vasoproliferation. qRT-PCR assays showed that the levels of tsRNA-1599 expression were significantly up-regulated in the OIR retinas (Figure 2D).

Diabetic retinopathy is considered a microvascular complication of diabetes 23. STZ was administered to 8-week-old C57BL/6J mice over 5 consecutive days to build the diabetic murine model. Retinal tissues were extracted at 4 months following the induction of diabetes. The levels of tsRNA-1599 expression were significantly up-regulated in diabetic retinas compared with the non-diabetic control (Figure 2E). Moreover, the expression levels of tsRNA-1599 were found to be up-regulated in HUVECs in response to both high glucose stress and oxidative stress (Figure S1A-B). To reveal the clinical relevance of tsRNA-1599 with neovascular diseases, we collected aqueous humor (AH) samples from nAMD, DR, and age-related cataract (ARC) patients. qRT-PCR assays revealed that the level of tsRNA-1599 was significantly higher compared to that of ARC patients (Figure 2F). Collectively, these results suggest that tsRNA-1599 expression is significantly up-regulated in neovascularization *in vitro* and *in vivo*.

### tsRNA-1599 regulates endothelial angiogenic effects *in vitro*

**Figure 3:**
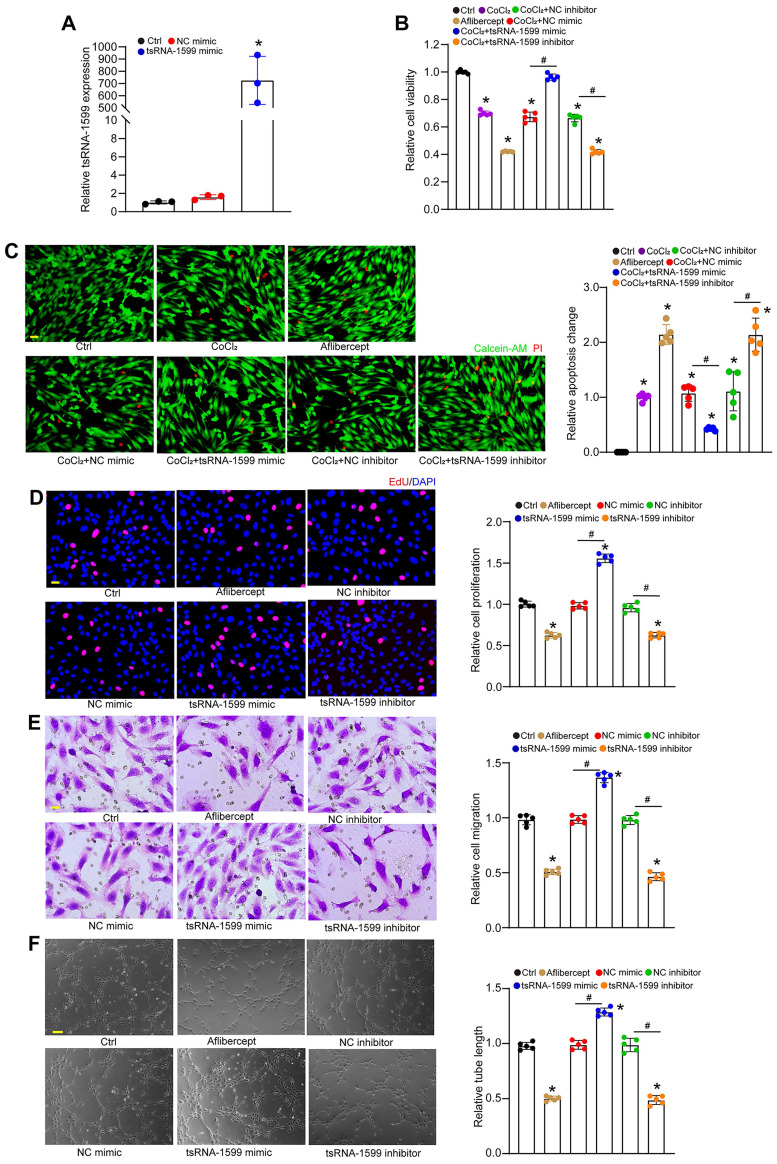
** tsRNA-1599 regulates endothelial angiogenic effects *in vitro*.** (A) HUVECs were transfected with negative control (NC) mimic, tsRNA-1599 mimic, or left untreated (Ctrl) for 24 h. The levels of tsRNA-1599 expression were detected by qRT-PCRs (n = 3, *P < 0.05 vs. Ctrl group, One-way ANOVA followed by Bonferroni's post hoc test). (B and C) HUVECs were transfected with NC mimic (30 nM), tsRNA-1599 mimic (30 nM), NC inhibitor (30 nM, tsRNA-1599 inhibitor (30 nM), treated with aflibercept (40 μg/mL), or left untreated (Ctrl) for 24 h, and then treated with or without CoCl_2_ (300 μmol/L) for 24 h. The viability of HUVECs was determined by CCK-8 assays (B, n = 5). Calcein-AM/PI assays were conducted to detect cell apoptosis (C, n = 5, Scale bar, 20 μm). *P < 0.05 vs. Ctrl group; ^#^*P* < 0.05 between the marked group; One-way ANOVA followed by Bonferroni's post hoc test. (D - F) HUVECs were transfected with NC mimic, tsRNA-1599 mimic, NC inhibitor, tsRNA-1599 inhibitor, treated with aflibercept (40 μg/mL), or left untreated (Ctrl) for 24 h. The proliferation ability of HUVECs was determined by EdU assays (D, n = 5, Scale bar, 20 μm). Cell migration and quantitative analysis was conducted by transwell assays (E, n = 5, Scale bar, 20 μm). Tube formation assays and quantitative analysis were conducted to detect the tube formation ability of HUVECs (F, n = 5, Scale bar, 50 μm). *P < 0.05 vs. Ctrl group; ^#^*P* < 0.05 between the marked group; One-way ANOVA followed by Bonferroni's post hoc test.

To determine the role of tsRNA-1599 in endothelial angiogenic effects *in vitro*, we regulated the levels of tsRNA-1599 expression by transfecting tsRNA-1599 mimic and inhibitor into HUVECs or HRVECs. qRT-PCR assays revealed that transfection of tsRNA-1599 mimic obviously enhanced the levels of tsRNA-1599 expression (Figure 3A and S2A). We first determined the role of tsRNA-1599 in the regulation of endothelial cell viability by CCK-8 assays. Compared with the control group, CoCl_2_ or high glucose treatment led to reduced endothelial cell viability. Transfection of tsRNA-1599 mimic could reverse CoCl_2_ or high glucose-induced reduction of cell viability. By contrast, transfection of tsRNA-1599 inhibitor could further decrease cell viability, showing a similar effect as aflibercept in inhibiting cell viability (Figure 3B and S2B). We further investigated the role of tsRNA-1599 in cell apoptosis. Calcein-AM/PI staining showed that the percentage of PI-positive cells obviously increased following CoCl_2_ or high glucose treatment. Transfection of tsRNA-1599 mimic led to reduced apoptosis as shown by decreased number of PI positive cells. By contrast, transfection of tsRNA-1599 inhibitor aggravated cell apoptosis, showing a similar effect as aflibercept in increasing cell apoptosis (Figure 3C and S2C).

Moreover, we determined the role of tsRNA-1599 in endothelial angiogenic effects by detecting endothelial proliferation, migration, and tube formation ability. Compared with the control group, transfection of tsRNA-1599 mimic led to increased cell proliferation, whereas transfection of tsRNA-1599 inhibitor led to a marked reduction of cell proliferation (Figure 3D and S2D). Transwell assays showed that the number of migrated cells in tsRNA-1599 mimic-transfected group was significantly greater than that in the control group, while transfection of tsRNA-1599 inhibitor obviously reduced the migration ability of endothelial cells (Figure 3E and S2E). Matrigel assays were conducted to determine the role of tsRNA-1599 in vascular tube formation. An obvious increase in tube formation ability was detected in tsRNA-1599 mimic-transfected group, while transfection of tsRNA-1599 inhibitor led to a marked reduction of tube formation ability (Figure 3F and S2F). Notably, tsRNA-1599 inhibitor displayed similar anti-angiogenic effects as aflibercept in endothelial cells. Collectively, the above-mentioned results suggest that tsRNA-1599 has emerged as a critical regulator of endothelial angiogenic effects *in vitro*.

### tsRNA-1599 silencing plays an anti-angiogenic role in experimental angiogenesis

**Figure 4:**
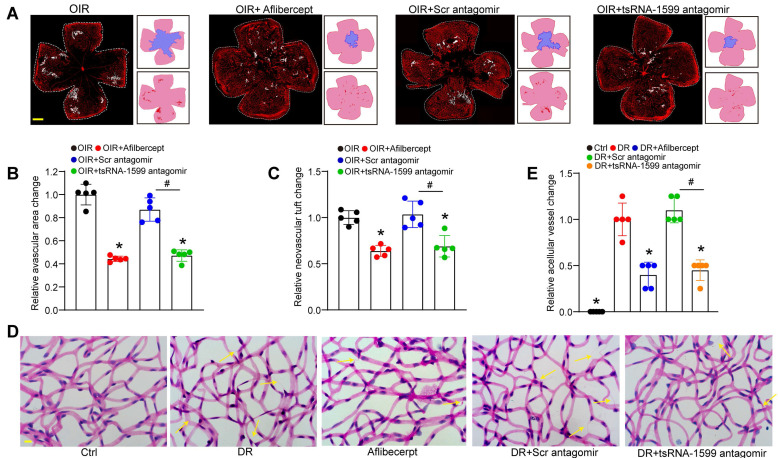
** tsRNA-1599 silencing plays an anti-angiogenic role in experimental angiogenesis.** (A) IB4 staining of the whole-mount retinas from OIR mice injected without or with aflibercept (40 mg/mL, 1 μL), scramble (Scr) antagomir (20 μM, 1 μL), or tsRNA-1599 antagomir (20 μM, 1μL) at P17 (n = 5 mice for each group, Scale bar, 500 μm) along with the quantification of avascular areas and neovascular tufts (NVTs). White line denotes retinal margin and white area represents NVT. In the insets, red line: retinal margin; blue area: avascular area; red area: NVTs. (B and C) Quantification analysis of avascular areas and NVTs, respectively. *P < 0.05 vs. OIR group; ^#^*P* < 0.05 between the marked group; One-way ANOVA followed by Bonferroni's post hoc test. (D and E) Retinal trypsin digestion was conducted to detect the number of acellular capillaries in non-DR mice (Ctrl), DR mice, DR mice-injected Scr antagomir (20 μM, 2 μL), tsRNA-1599 antagomir (20 μM, 2 μL), or aflibercept (40 mg/mL, 2 μL). Yellow arrows indicated acellular capillaries (n = 5 mice for each group, Scale bar, 10 μm). *P < 0.05 vs. DR group;^ #^*P* < 0.05 between the marked group; One-way ANOVA followed by Bonferroni's post hoc test.

Due to the critical role of tsRNA-1599 in endothelial angiogenic effects *in vitro*, we then determine the role of tsRNA-1599 in ocular neovascularization *in vivo*. We first used the OIR model that can mimic ocular angiogenic features and investigated the role of tsRNA-1599 in retinal angiogenesis. The avascular areas and neovascular tuft (NVT) areas in the mice of tsRNA-1599 antagomir-injected group were obviously reduced compared to that in the control group (Figure 4A-C), whereas injection of tsRNA-1599 agomir displayed the pro-angiogenic effects on retinal angiogenesis (Figure S3A-C). STZ-induced diabetic model was then used to determine the role of tsRNA-1599 in retinal vascular dysfunction. Trypsin digestion assays revealed that injection of tsRNA-1599 antagomir could ameliorate capillary degeneration in diabetic mice as shown by decreased number of acellular capillaries (Figure 4D-E), while tsRNA-1599 overexpression could increase the number of acellular capillaries (Figure S3D-E). Notably, tsRNA-1599 antagomir exhibited comparable anti-angiogenic effects as aflibercept in ocular angiogenesis.

CNV model was also used to determine the role of tsRNA-1599 in ocular angiogenesis on day 14 following laser injury. qRT-PCR assays showed that injection of tsRNA-1599 agomir significantly increased the levels of tsRNA-1599 in the choroid/RPE tissues, while tsRNA-1599 antagomir decreased the levels of tsRNA-1599 (Figure S4A). Quantification of CNV lesions revealed that injection of tsRNA-1599 antagomir led to about 50% reduction of CNV lesion areas, showing a similar anti-angiogenic effect as aflibercept. By contrast, injection of tsRNA-1599 agomir led to about 60% increase in CNV lesions (Figure S4B-C).

An *ex vivo* model of choroidal sprouting is an important model to study choroidal neovascularization 24. We used the choroidal sprouting model to examine the role of tsRNA-1599 in angiogenesis. Angiogenic areas occupied by the migrated cells were detected on day 4, day 5, and day 6 following choroid/RPE seeding. Compared with the control group, sprouting areas from the choroidal explants reduced following tsRNA-1599 silencing by transfection of tsRNA-1599 antagomir, but increased following the transfection of tsRNA-1599 agomir. Moreover, tsRNA-1599 antagomir had a similar anti-angiogenic effect as aflibercept in inhibiting choroidal sprouting (Figure S4D-E). Together, these results suggest that tsRNA-1599 silencing plays an anti-angiogenic role in experimental angiogenesis.

### tsRNA-1599 delivery has no obvious retinal toxicity *in vivo*

**Figure 5:**
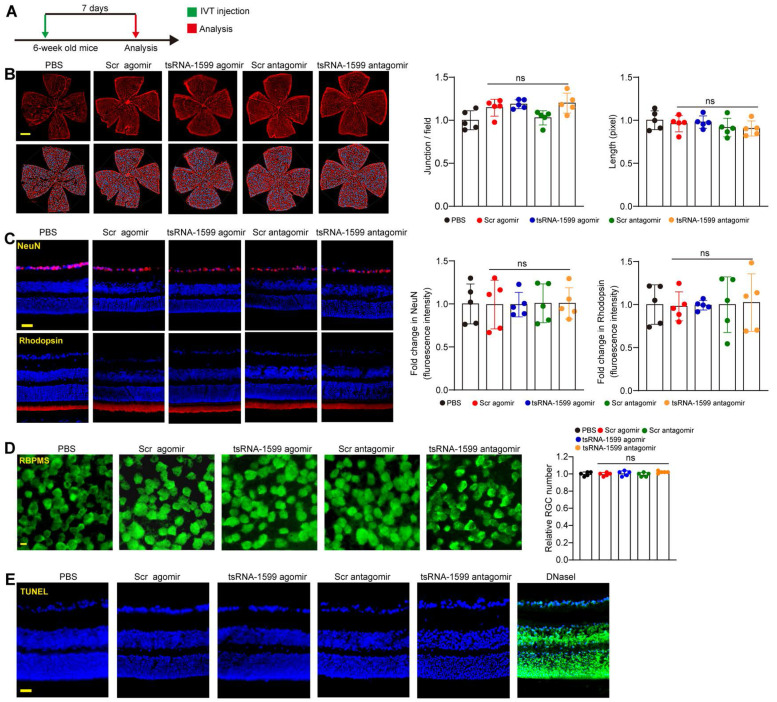
** Delivery of tsRNA1599 has no obvious retinal toxicity *in vivo*.** (A) Diagram illustrating the experimental procedure for assessing the effects of altered tsRNA-1599 levels on the retinal toxicity for 7 days. (B) IB4 staining of retinal flat-mounts injected with scramble (Scr) agomir (20 μM, 2 μL), tsRNA-1599 agomir (20 μM, 2 μL), Scr antagomir (20 μM, 2 μL), tsRNA-1599 antagomir (20 μM, 2 μL), or PBS for 7 days (Scale bar, 500 μm). Quantification of vascular junction and length were shown (n = 5). (C) Immunofluorescence staining of the retinas injected with Scr agomir (20 μM, 2 μL), tsRNA-1599 agomir (20 μM, 2 μL), Scr antagomir (20 μM, 2 μL), tsRNA-1599 antagomir (20 μM, 2 μL), or PBS for 7 days with NeuN and Rhodopsin (Scale bar, 50 μm). Quantification results and representative images of NeuN and Rhodopsin staining were shown (n = 5). (D) The retinas were administered with Scr agomir (20 μM, 2 μL), tsRNA-1599 agomir (20 μM, 2 μL), Scr antagomir (20 μM, 2 μL), tsRNA-1599 antagomir (20 μM, 2 μL), or PBS for 7 days (Scale bar, 20 μm). Quantitative results and representative images of RBPMS staining are depicted (n = 5). The displayed images were captured at a location halfway between the center and the periphery of retina. (E) TUNEL staining of the retinas injected with Scr agomir (20 μM, 2 μL), tsRNA-1599 agomir (20 μM, 2 μL), Scr antagomir (20 μM, 2 μL), tsRNA-1599 antagomir (20 μM, 2 μL), or PBS for 7 days (Scale bar, 50 μm). “ns” represents no statistical significance; One-way ANOVA followed by Bonferroni's post hoc test.

The above-mentioned results indicate that tsRNA-1599 is a critical regulator of ocular neovascularization. We next determined whether altered tsRNA-1599 expression had the detrimental effects on the existing vessels, RGCs, and photoreceptors in the retinas. Injection of tsRNA-1599 agomir and antagomir did not destroy the existing vessels compared with PBS group at day 7 following the injection (Figure 5A-B). We extended these findings by injecting the adult mice with tsRNA-1599 agomir and antagomir for 14 days and 28 days. The results show that long-term injections of tsRNA-1599 agomir and antagomir had no obvious detrimental effects on the existing mature vessels (Figure S5).

Subsequently, we performed the immunofluorescence staining to determine the effects of tsRNA-1599 delivery on RGC survival and photoreceptor degeneration by the injections of tsRNA-1599 agomir and antagomir for 7 days, 14 days and 28 days. Compared with PBS group, altered tsRNA-1599 level had no obvious detrimental effects on RGC survival and photoreceptor degeneration as shown by unaltered fluorescence intensity of NeuN-positive RGCs and Rhodopsin-positive photoreceptors (Figure 5C and Figure S6). Additionally, RBPMS staining was conducted to detect RGC survival. The results demonstrated that intravitreal injection of tsRNA-1599 agomir and antagomir had no obvious detrimental effects on RGC survival (Figure 5D and Figure S7A-B). TUNEL assays further verified that altered tsRNA-1599 level did not cause a detectable apoptosis in retinal tissues (Figure 5E and Figure S7C-D). Together, these results indicate that injections of tsRNA-1599 agomir and antagomir do not exhibit significant detrimental effects on pre-existing vessels, RGCs, and photoreceptors.

### tsRNA-1599 is an indirect regulator of VEGF signaling

**Figure 6:**
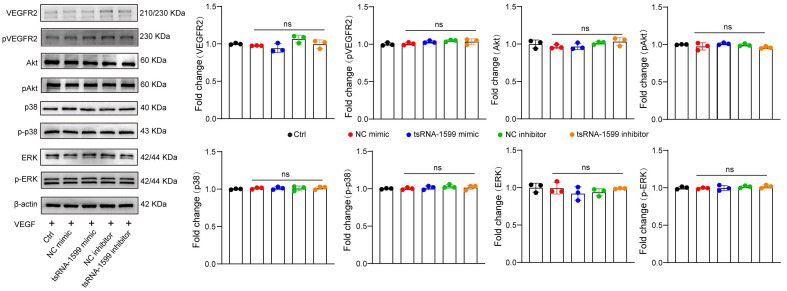
** tsRNA-1599 is an indirect regulator of VEGF signaling in endothelial cells.** HUVECs were transfected with negative control (NC) mimic (30 nM), tsRNA-1599 mimic (30 nM), NC inhibitor (30 nM, tsRNA-1599 inhibitor (30 nM), or left untreated (Ctrl) for 24 h, and then stimulated with VEGF (100 ng/mL) for up to 2 h. Western blots were conducted to detect the expression levels of VEGFR2, p-VEGFR2, Akt, p-Akt, p38, p-p38, ERK, and p-ERK (n = 3). β-actin was detected as the internal control. “ns” represents no statistical significance; One-way ANOVA followed by Bonferroni's post hoc test.

Previous studies have revealed that VEGF is a key driver of pathological angiogenesis and anti-VEGF drugs are the major strategy for anti-angiogenic treatment 25. We thus evaluated whether VEGF signaling was affected following tsRNA-1599 delivery. HUVECs were treated in the presence of VEGF, followed by the transfection of tsRNA-1599 mimic and inhibitor. Western blot analysis demonstrated that transfection of tsRNA-1599 mimic and inhibitor had no effects on VEGFR2 phosphorylation at Y1175 and did alter the phosphorylated levels of Akt, ERK, and p38 in response to VEGF stimulation (Figure 6). Additionally, we also compared the effect between tsRNA-1599 antagomir group and aflibercept plus tsRNA-1599 antagomir group to investigate whether the pro-angiogenic properties of tsRNA-1599 were truly VEGF independent in CNV, OIR and DR models. As was shown in the Figure S8, combination of tsRNA-1599 antagomir with aflibercept did not show an additive effect on ocular neovascularization *in vivo*. This lack of additive effects between VEGF and tsRNA-1599 on retinal vascular development may reflect that tsRNA-1599 and VEGF still share common downstream cellular events in endothelial cells that are limiting or tightly regulated. These results indicate that transfection of tsRNA-1599 agomir and antagomir had no effect on the events of VEGF-induced phosphorylation of VEGFR downstream signaling, suggesting that tsRNA-1599 appears to be an indirect regulator of VEGF signaling in endothelial cells.

### tsRNA-1599 regulates glycolytic balance in endothelial cells

To investigate the potential mechanism of tsRNA-1599 in endothelial angiogenic effects, transcriptomic analysis was conducted using HUVEC-transfected with tsRNA-1599 inhibitor or negative control inhibitor for 24 h (Figure S9A). Compared with NC group, a total of 309 differentially expressed genes (Log_2_FC ≥ ±1; *P* value ≤ 0.05) were identified in tsRNA-1599 inhibitor-transfected group, including 149 up-regulated and 160 down-regulated genes (Figure S9B). Gene set enrichment analysis (GSEA) was performed to search for the underlying function of tsRNA-1599 in endothelial cells. tsRNA-1599 silencing led to a marked down-regulation of the signaling pathways involved in glycolysis and MYC_target signaling in HUVECs, and an obvious up-regulation of pentose_phosphate pathway (Figure S9C).

**Figure 7:**
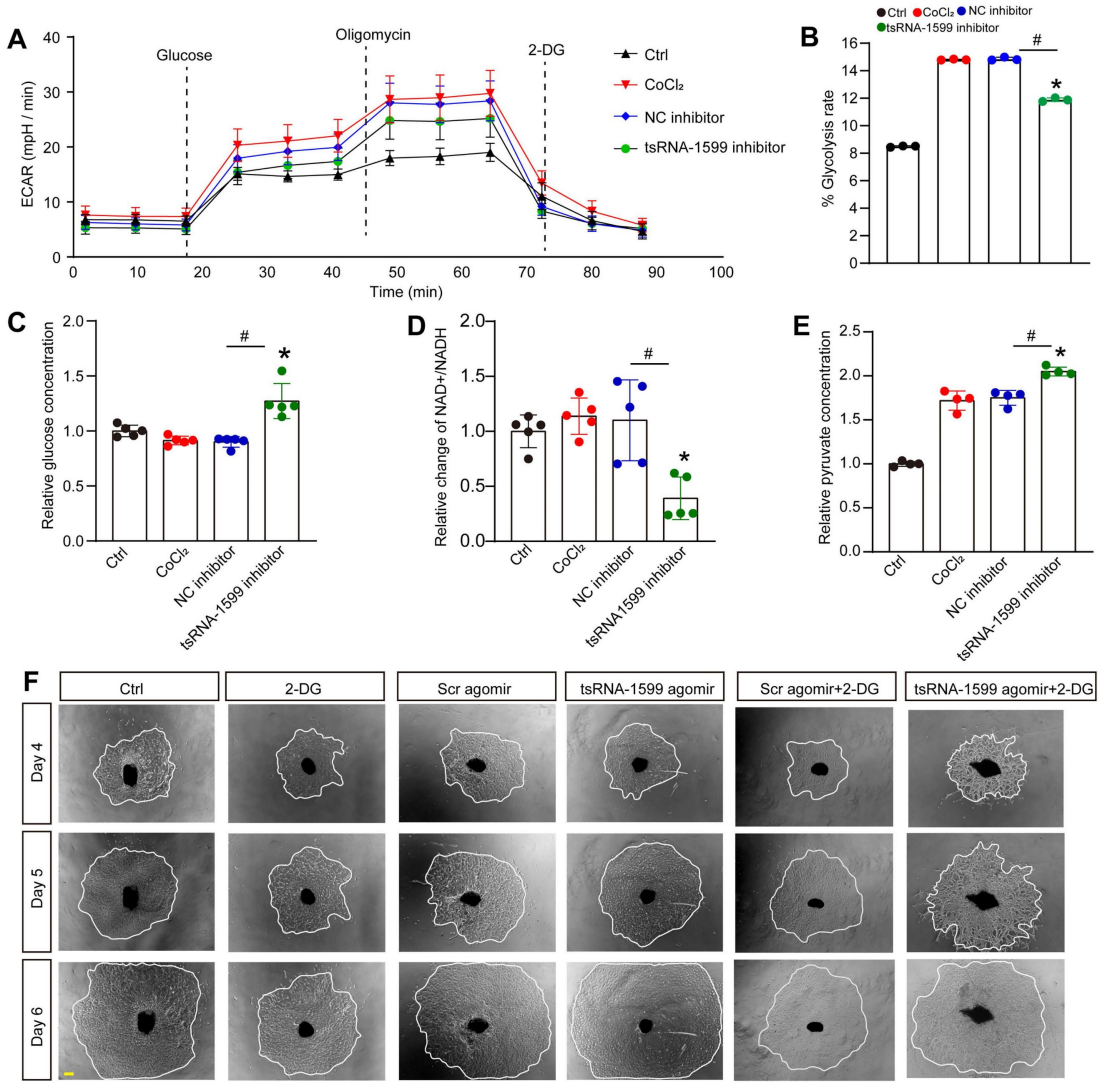
**tsRNA-1599 regulates glycolytic balance in endothelial cells.** (A-E) HUVECs were transfected with negative control (NC) inhibitor (30 nM), tsRNA-1599 inhibitor (30 nM), or left untreated, and the exposed to CoCl_2_ (300 μmol/L) to mimic hypoxic condition for 24 h. The group cultured in normal condition was taken as Ctrl group. Seahorse analysis of glycolysis (ECAR) was conducted at 24 h following treatment (A). The concentration of reagents used in ECAR assays was as followed: glucose (10 mM), oligomycin (2 μM), and 2‐deoxyglucose (2‐DG, 100 mM). ECAR analysis was conducted in HUVECs following tsRNA-1599 inhibition (B, n = 3, *P < 0.05 vs. Ctrl group; ^#^*P* < 0.05 between the marked group; Kruskal-Wallis's test followed by Bonferroni's post hoc test). Glucose levels in culture medium were detected following tsRNA-1599 inhibition (C, n = 5, *P < 0.05 vs. Ctrl group; ^#^*P* < 0.05 between the marked group; Kruskal-Wallis's test followed by Bonferroni's post hoc test). NAD^+^/NADH ratio was determined in HUVECs following tsRNA-1599 inhibition and the absorbance was measured at 450 nm (D, n = 5, *P < 0.05 vs. Ctrl group; ^#^*P* < 0.05 between the marked group; Kruskal-Wallis's test followed by Bonferroni's post hoc test). Pyruvate level was determined in HUVECs following tsRNA-1599 inhibition and the absorbance was measured at 520 nm (E, n = 4, *P < 0.05 vs. Ctrl group; ^#^*P* < 0.05 between the marked group; One-way ANOVA followed by Bonferroni's post hoc test). (F) Representative images of choroidal explants cultured in the presence or absence of tsRNA-1599 agomir with or without 2-DG (50 mM). The sprouting potency of choroidal explants were photographed on day 4, day 5, and day 6 (Scale bar, 500 μm).

To further determine the change of endothelial metabolism following tsRNA-1599 expression change, we measured the extracellular acidification rate (ECAR), an indicator of glycolysis (Figure 7A). Transfection of tsRNA-1599 inhibitor led to a marked reduction of ECAR compared with NC inhibitor group, suggesting that inhibition of tsRNA-1599 decreased glycolytic capacity (Figure 7B). Given the reduction in glycolytic flux observed in HUVECs, an experiment was conducted to determine if glucose uptake was altered following the transfection of tsRNA-1599 inhibitor. Transfection of tsRNA-1599 inhibitor led to reduced glucose uptake as shown by the highest glucose concentration in culture medium in tsRNA-1599 inhibitor-transfected HUVECs (Figure 7C). NAD^+^/NADH redox couple serves as a regulator of cellular energy metabolism, including glycolysis and mitochondrial oxidative phosphorylation. Transfection of tsRNA-1599 inhibitor led to a decreased NAD^+^/NADH ratio (Figure 7D). Accordingly, a marked increase in intracellular accumulation of pyruvate was observed following the transfection of tsRNA-1599 inhibitor (Figure 7E). The addition of tsRNA-1599 agomir resulted in a marked increase in choroidal sprouting areas. The addition of 2-DG (2-Deoxyglucose, a potent inhibitor of glycolysis) abrogated the pro-angiogenic effects of tsRNA-1599 agomir on choroidal sprouting, suggesting that glycolysis plays a significant role in ocular angiogenesis (Figure 7F). Collectively, the aforementioned data suggests that the administration of tsRNA-1599 inhibitor leads to an accumulation of pyruvate and a decrease in NAD^+^ regeneration.

### tsRNA-1599 regulates endothelial angiogenic effects by interacting with YBX1

**Figure 8:**
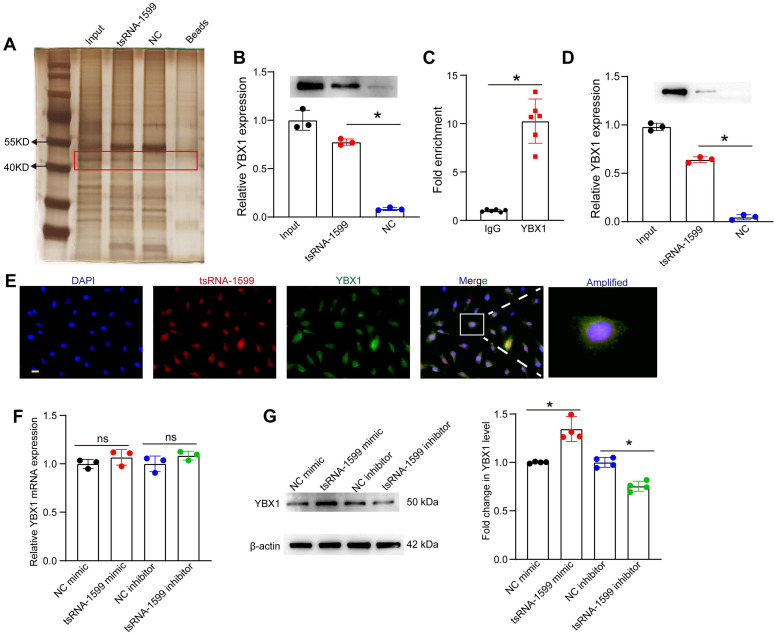
** tsRNA-1599 regulates endothelial angiogenic effects by interacting with YBX1.** (A) Silver staining of tsRNA-1599-associated proteins following RNA pull-down assays by tsRNA-1599 mimic, scramble tsRNA mimic (NC), and streptavidin beads. (B) Western blot analysis of YBX1 expression following RNA pull-down assays using tsRNA-1599 mimic or scramble tsRNA mimic (NC) (n = 3, *P < 0.05 between the marked group, One-way ANOVA followed by Bonferroni test). (C) RIP-qPCR analysis of tsRNA-1599 expression following RNA immunoprecipitation using anti-YBX1 or IgG in HUVECs (n = 6, *P < 0.05 between the marked group, Student *t* test). (D) Western blot analysis of YBX1 expression following RNA pull-down assays using tsRNA-1599 agomir or scramble tsRNA agomir (NC) in RPE-choroid-sclera complex (n = 3, *P < 0.05 between the marked group, One-way ANOVA followed by Bonferroni test). (E) Immunostaining assays and RNA-FISH assays were conducted to detect the expression of tsRNA-1599 (red) and YBX1 (green) in HUVECs. (F and G) HUVECs were transfected with NC mimic, tsRNA-1599 mimic, NC inhibitor, or tsRNA-1599 inhibitor for 24 h. qPCR assays (F, n = 3) and western blots (G, n = 4) were conducted to detect the expression of YBX1. *P < 0.05 between the marked group; “ns” indicates no statistical significance; Student *t* test.

Because tsRNA-1599 was mainly expressed in the nuclei of HUVECs, we speculated that tsRNA-1599 played its role at the transcriptional level. To explore the underlying mechanism of tsRNA-1599-mediated endothelial angiogenic effects, the endogenous binding proteins of tsRNA-1599 were identified by RNA pull-down assays in HUVECs. The binding proteins were shown by silver staining. Compared with the control group, a stronger band at ~50 kDa was detected in tsRNA-1599 mimic-transfected group (Figure 8A). We further analyzed the binding proteins by mass spectrometry (Table S1). We found that the top-ranked Y-box binding protein 1 (YBX1) was tightly associated with neovascularization and glycolysis. As a DNA and RNA binding protein, YBX1 is potentially involved in both transcriptional and post-transcriptional gene regulation 26. The interaction was validated by western blots using anti-YBX1 antibody (Figure 8B). We further verified the interaction between tsRNA-1599 and YBX1 by RIP-qPCR assays and found that tsRNA-1599 was highly enriched in the immunoprecipitates by YBX1 but not IgG (Figure 8C). To validate the interaction between tsRNA-1599 and YBX1 *in vivo*, pull down and western blots assays were conducted using anti-YBX1 antibody on RPE-choroid-sclera complexes. The results revealed that compared to Scr tsRNA group, YBX1 was enriched in tsRNA-1599 group, indicating the tsRNA-1599 could bind to YBX1 protein *in vivo* (Figure 8D). Moreover, immunostaining and FISH assays verified the co-localization between tsRNA-1599 and YBX1 protein in the nuclei (Figure 8E). Subsequently, qRT-PCR assays and western blots were used to detect whether tsRNA-1599 regulated the expression levels of YBX1. tsRNA-1599 did not alter the levels of YBX1 mRNA but affected the levels of YBX1 protein. The levels of YBX1 protein expression were significantly down-regulated following transfection of tsRNA-1599 inhibitor. By contrast, the levels of YBX1 protein expression were up-regulated following tsRNA-1599 overexpression (Figure 8F-G). Collectively, these data suggests that tsRNA-1599 regulates endothelial angiogenic effects by interacting with YBX1.

### tsRNA-1599 regulates endothelial glycolytic metabolism by targeting HK2

**Figure 9:**
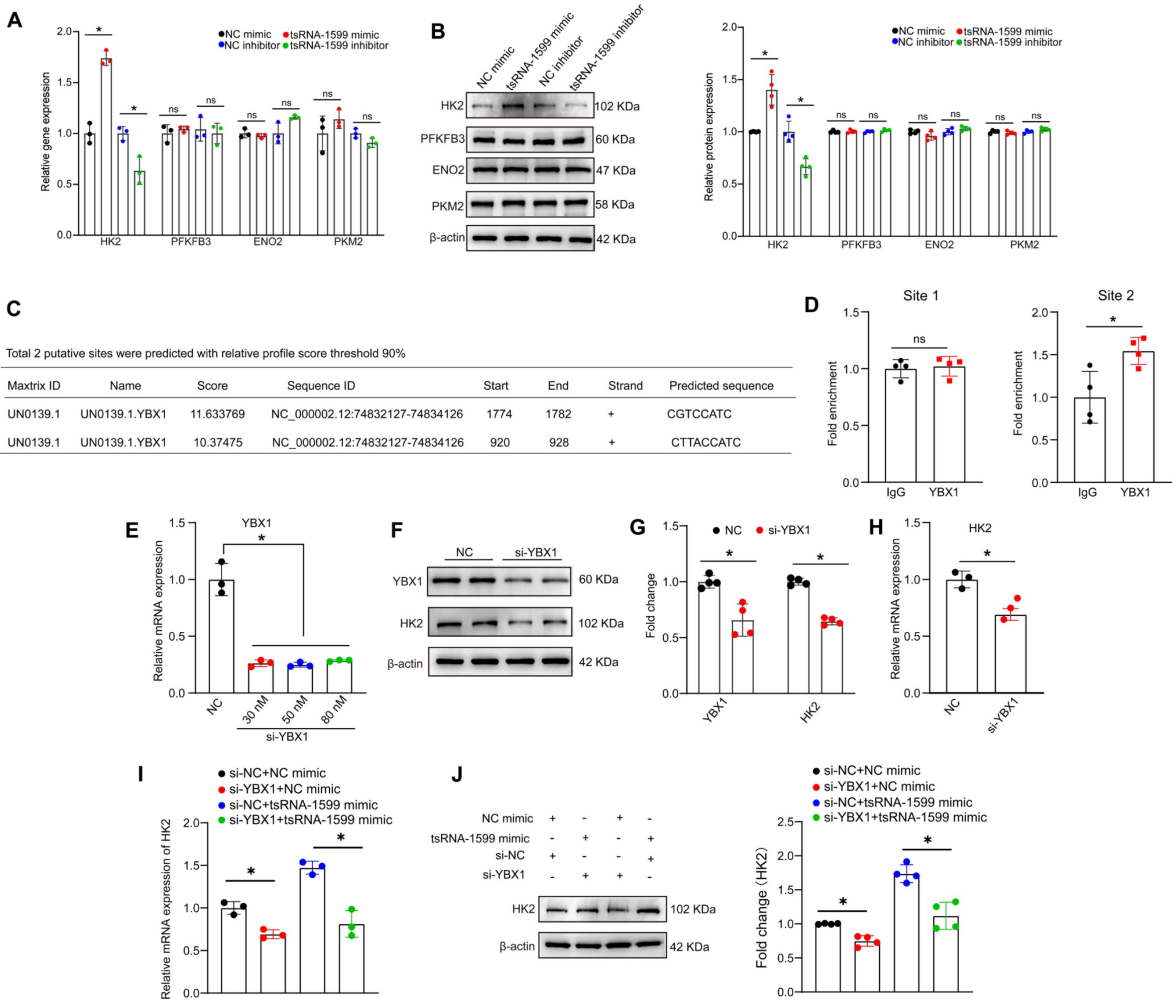
** tsRNA-1599 regulates HK2 expression via interacting with YBX1.** (A-B) HUVECs were transfected with negative control (NC) mimic (30 nM), tsRNA-1599 mimic (30 nM), NC inhibitor (30 nM), or tsRNA-1599 inhibitor (30 nM) for 48 h. qPCR assays (A, n = 3) and western blots were conducted to detect the expression of selected genes (B, n = 4). *P < 0.05 between the marked group; Student *t* test; “ns” represents no statistical significance. (C) The predicted result of binding sites between YBX1 and HK2 on the JASPAR website. (D) ChIP-qPCR results of the predicted binding sites (n = 4, *P < 0.05 between the marked group; “ns” represents no statistical significance; Student *t* test). (E - H) HUVECs were transfected with NC siRNA or YBX1 siRNA for 24 h. qPCR assays (E, n = 3, One-way ANOVA followed by Bonferroni's post hoc test, Scale bar, 20 μm) and western blots were conducted to detect the expression of YBX1 or HK2 (F - G, n = 4, Student *t* test, *P < 0.05 between the marked group). (H) qPCR assays were used to verify the expression of HK2 (n = 3, *P < 0.05 vs. NC group, Student *t* test). (I - J) HUVECs were transfected with negative control (NC) mimic (30 nM) plus NC siRNA (30 nM), NC mimic (30 nM) plus YBX1 siRNA (30 nM), NC siRNA (30 nM) plus tsRNA-1599 mimic (30 nM), YBX1 siRNA (30 nM) plus tsRNA-1599 mimic (30 nM) for 48 h. qPCR assays (I, n = 3) and western blots (J, n = 4) were conducted to detect the expression of HK2. *P < 0.05 between the marked group, Student *t* test.

Since tsRNA-1599 is a critical regulator of endothelial glycolytic metabolism, we thus selected four glycolysis-related genes from RNA sequencing results to verify their expression pattern. qRT-PCR assays and western blots revealed that the expression of hexokinase 2 (HK2) gene was significantly up-regulated following the transfection of tsRNA-1599 mimic, whereas HK2 expression was down-regulated following tsRNA-1599 silencing. However, the levels of PFKFB3, ENO2, and PKM2 expression were not altered (Figure 9A-B). We selected HK2 gene for further study due to its critical role in the occurrence and progression of neovascular diseases 27-29. Subsequently, we designed three different HK2 siRNAs to reduce its expression (Figure S10A-B). EdU assays revealed that the transfection of tsRNA-1599 mimic resulted in increased cell proliferation. In contrast, transfection of HK2 siRNA reversed the increased proliferation induced by the overexpression of tsRNA-1599 (Figure S10C-D). Transwell assays showed that tsRNA-1599 overexpression increased the number of migrated cells compared with the control group and HK2 knockdown curtailed increased migration caused by tsRNA-1599 overexpression (Figure S10E-F). Matrigel assays demonstrated an increased tube formation ability when tsRNA-1599 was overexpressed, and the downregulation of HK2 interrupted increased tube formation induced by tsRNA-1599 overexpression (Figure S10G-H). Collectively, these findings suggest that tsRNA-1599 regulates endothelial angiogenic process through the regulation of HK2.

We further investigated whether tsRNA-1599 regulated HK2 gene expression via YBX1. Utilizing the open-access database JASPAR, we predicted the potential binding sites between YBX1 and HK2, revealing two sites meeting the criteria of a 90% relative profile score threshold (Figure 9C). ChIP-qPCR assays revealed that only site2 likely became the binding site (Figure 9D). To validate the impact of YBX1 on HK2 expression, we performed YBX1 knockdown experiments, observing a downregulation of HK2 at both mRNA and protein levels (Figure 9E-H). To explore the potential regulatory relationship among tsRNA-1599, YBX1, and HK2, we conducted simultaneous YBX1 knockdown and tsRNA-1599 overexpression experiments. The results demonstrated that overexpression of tsRNA-1599 can promote HK2 expression, while knockdown of YBX1 can partially rescue this promoting effect on HK2 expression (Figure 9I-J). Collectively, these results suggest that tsRNA-1599 regulates HK2 gene expression through YBX1, thereby influencing endothelial glycolytic metabolism.

## Discussion

Neovascularization has been implicated in the pathogenesis of ocular neovascular diseases. Current anti-angiogenic treatments are highly dependent on anti-VEGF drugs 30, 31. However, some patients with ocular angiogenesis develop the resistance to anti-VEGF drugs and the concerns of increased risk of neurotoxic effects 32. In this study, we have identified a novel inhibitor of angiogenesis, tsRNA-1599. Silencing of tsRNA-1599 plays an anti-angiogenic role *in vivo* and *in vitro*. Altered tsRNA-1599 expression has no obvious detrimental effects on the existing mature vessels, RGCs, and photoreceptors. tsRNA-1599 has no direct effects on VEGF signaling but can alter glycolysis and NAD^+^/NADH production in endothelial cells. Collectively, tsRNA-1599 is a key regulator of pathological ocular angiogenesis and targeting tsRNA-1599-mediated signaling is a promising method for treating ocular neovascular diseases.

tsRNAs are a novel type of non-coding small RNAs derived from the precursor or the mature tRNAs, which play important roles in the occurrence and progression of human diseases 33, 34. Nonetheless, tsRNA profile in ocular neovascular disease is still unknown. In this study, we first sequenced RPE-choroid-sclera complexes from the mice with laser-induced CNV and the corresponding controls to identify neovascularization-related tsRNAs. Intriguingly, tsRNA-1599 was markedly upregulated in animal models of ocular angiogenesis and endothelial cells responding to pro-angiogenic stress. tsRNA-1599 is a specific tRNA half derived from the T-loop of mature tRNA-Tyr-GTA and is primarily localized in the nuclei. Silencing of tsRNA-1599 can reduce cell viability, proliferation, migration, and tube formation. By contrast, overexpression of tsRNA-1599 can enhance cell viability, proliferation, migration, and tube formation ability. Moreover, delivery of tsRNA-1599 antagomir can suppress pathological ocular neovascularization *in vivo*. In our previous study, we identified another tRF, tRF-1001, which is also involved in pathological ocular angiogenesis. The levels of tRF-1001 expression are decreased in the retinas of OIR model, choroidal neovascularization model, and endothelial sprouting model. Tip-stalk cell specification is an early event of sprouting angiogenesis. Increased tRF-1001 suppresses ocular angiogenesis by targeting tip-stalk endothelial specialization. Mechanistically, silencing of tRF-1001 leads to increased expression of METTL3, which can repress the expression of RBPJ and MAML1 through an YTHDF2-dependent mechanism acting on their mRNAs. Collectively, the evidence suggests that targeting signaling mediated by tRNA-derived small RNAs represents a promising therapeutic approach for ocular vascular diseases 19.

We also explored the mechanism by which tsRNA-1599 contributes to ocular angiogenesis. Previous studies have revealed the involvement of tsRNAs in several human diseases, such as neurodegenerative disorders, metabolic diseases, and cancers 35-37. tsRNA can regulate cellular processes via distinct molecular mechanisms, such as gene silencing, translational reprogramming, and competitive binding to essential proteins 38, 39. VEGF signaling is established as a key driver of angiogenesis. It has been identified as a major therapeutic target for anti-angiogenic treatment 40. We investigated the effects of altered tsRNA-1599 expression on VEGF signaling by detecting levels of key signaling proteins. Delivery of tsRNA-1599 did not directly alter VEGF signaling, suggesting that its effects on angiogenesis in endothelial cells appear to be an indirect consequence of altered VEGF signaling. We revealed that the combination of a tsRNA-1599 antagomir with aflibercept had no additive effect on ocular angiogenesis, implying that the pro-angiogenic effect of tsRNA-1599 is not entirely VEGF-independent. This lack of additive effects between VEGF and tsRNA-1599 on retinal vascular development may reflect shared downstream cellular events. Additionally, tsRNA-1599 likely acts on non-vascular cells in the retina, which may indirectly mask the effects of anti-VEGF treatment on the retinal endothelium. The role of tsRNA-1599 in neurotrophic effects or its impact on non-vascular retinal cells warrants further investigation.

One of the interesting findings is the observation that tsRNA-1599 regulates energy metabolism in endothelial cells. We performed RNA sequencing to investigate the potential mechanism underlying angiogenic effects of tsRNA-1599 and revealed that it altered the glycolytic balance in endothelial cells. Several studies have highlighted the importance of metabolic regulation in endothelial cells and unveiled the key role of glycolytic pathway in angiogenic process 41-43. In this study, administration of a tsRNA-1599 inhibitor caused an accumulation of pyruvate and a decline in NAD^+^ regeneration, which can disrupt redox homeostasis and decrease ATP synthesis. ATP is a critical driver of endothelial cell activity by enhancing filopodia formation. Delivery of tsRNA-1599 inhibitor reduced endothelial metabolic activity, which may lead to a quiescent phalanx cell-like phenotype. Thus, inhibition of glycolytic flux mediated by tsRNA-1599 may ultimately inhibit pathological angiogenesis.

Previous studies have demonstrated that tsRNAs play their roles via interacting with some proteins 44, 45. In this study, RNA pull-down assays revealed that tsRNA-1599 can interact with the transcription factor YBX1. RIP and FISH assays further confirmed the interaction between tsRNA-1599 and YBX1. YBX1 has broad nucleic acid-binding properties and has been implicated in several cellular processes, such as regulation of transcription and translation, pre-mRNA splicing, DNA repair, and mRNA packaging. YBX1 is localized to the mitochondrial intermembrane space by its C-terminal domain (CTD). In mitochondria, YBX1 can inhibit pyruvate uptake by associating with the mitochondrial pyruvate carriers MPC1/2, thereby suppressing pyruvate-dependent tricarboxylic acid (TCA) cycle flux. Additionally, YBX1 induces aerobic glycolysis by activating protein expression of HIF-1α and MYC in gastric cancer cells 46-48. We uncover that tsRNA-1599 governs the expression of YBX1. Intriguingly, while tsRNA-1599 doesn't impact the expression of YBX1 mRNA, it does influence its protein levels. Considering YBX1's involvement in pro-angiogenic processes, it's unsurprising that inhibiting tsRNA-1599 leads to a decrease in YBX1 expression, consequently exerting an anti-angiogenic effect.

## Conclusions

tsRNA-1599 is shown as a pro-angiogenic factor in ocular angiogenesis. This finding has great implications for the advancement of anti-angiogenic treatment. Specifically, we have identified a novel regulator of pathological angiogenesis that appears to indirectly affect VEGF signaling. Although we reveal that the effect of tsRNA-1599 is endothelial-specific, tsRNA-1599 may also play important roles in non-vascular retinal cells such as neural stem cells, ependymal cells, and photoreceptors. In addition, tsRNA-1599 plays its regulatory role in ocular angiogenesis via interacting with YBX1. As a DNA and RNA binding protein, YBX1 is potentially involved in both transcriptional and post-transcriptional gene regulation. It can participate in regulating the expression of numerous genes and bring about broad impacts beyond metabolic reprogramming. In the future, its manipulation safety should be concerned. Moreover, the role of tsRNA-1599 in neurotrophic effects are still required for further investigation.

## Methods

### Animal ethics statement

Animal experiments adhered to the guidelines set forth by the Association for Research in Vision and Ophthalmology (ARVO) regarding the use of animals in ophthalmic and vision research and approved by the Animal Care and Use Committee of the authors' institute (2020-03-30). C57BL/6J mice were procured from the Animal Core Facility of Nanjing Medical University in Nanjing, China. They were accommodated in a specific pathogen-free animal facility with a 12-hour light/12-hour dark cycle, maintained at a room temperature of 25 ± 1 ℃.

### Laser-induced choroidal neovascularization (CNV) model

Choroidal neovascularization was induced by laser photocoagulation with 532 nm wavelength, 50 μm spot size, 70 ms duration, and 130 mW power. Briefly, the mice were anesthetized by intraperitoneal injection of ketamine (80 mg/kg) and xylazine (10 mg/kg) and the pupils were dilated with 1% tropicamide eye drops (0.5%, Alcon, USA). Next, four spots at 3, 6, 9, and 12 o' clock position around optic discs were created in the posterior pole of retinas. A vaporization bubble without hemorrhage was deemed as successful. Following laser photocoagulation, the mice were placed on the heat lamp for recovery until they became awake. Two weeks after CNV induction, choroidal tissues were harvested for subsequent analysis 21.

### Oxygen-induced retinopathy model

C57BL/6J mouse pups were used for building oxygen-induced retinopathy (OIR) model 22. Briefly, the pups at the postnatal day 7 (P7) were exposed to 75% oxygen for 5 days. At P12, they were returned to normoxia (21% oxygen) for another 5 days. Relative hyperoxia (P7 to P12) induced vaso-obliteration, while relative hypoxia (P12 to P17) induced retinal neovascularization. The control mice were kept in room air. The retinas were enucleated at P17 for isolectin-B4 staining and observed under a fluorescence microscope (Olympus IX70, Tokyo, Japan).

### Streptozotocin (STZ)-induced diabetic retinopathy

After fasting for 12 h, C57BL/6J mice (male, 8-week old) received an intraperitoneal injection of streptozotocin (STZ, 50 mg/kg in citrate buffer, pH 4.5) or citrate buffer (Vehicle control) for 5 consecutive days. The levels of fasting blood glucose were determined at day 7 following STZ injection using a One Touch Ultra meter (Lifescan, CA). The blood glucose levels > 16.7 mmol/L were deemed diabetic. Sex- and age-matched normal C57BL/6J mice were used as the controls 23.

### Choroidal sprouting assay *ex vivo*

The eyes were enucleated and kept in the ice-cold Endothelial Cell Growth Medium-2 (EGM-2, Lonza, Cat. CC-3156). Retinal pigment epithelium (RPE)-choroid-sclera complex was isolated and dissected into 1 × 1 mm^2^ pieces. The pieces were carefully embedded in the growth factor-reduced Matrigel (BD Biosciences, USA, Cat. 354230). After Matrigel solidification, DMEM with 10% FBS (Gibco, USA, Cat. 10099141) and 1% penicillin/streptomycin (Gibco, USA, Cat. 15140122) was added and replaced. Choroidal sprouting was observed at day 4, day 5, and day 6 following seeding under 4 × magnification by Image J software 24.

### Choroid flat-mount isolectin-B4 staining

The eyes were enucleated and fixed in 4% paraformaldehyde (PFA; BL539A, Biosharp, China) for 1 h at room temperature. Then, the RPE-choroid-sclera complex was dissected, cut into four quadrants, and mounted on the glass slide. The choroids/retinas were blocked and permeabilized with 5% bovine serum albumin (BSA) and 1% Triton X-100 for 1 h at 37 ℃. After incubation with Isolectin-B4 overnight at 4 ℃, the flat-mount was observed by a fluorescence microscope (Olympus IX70, Tokyo, Japan).

### Retinal trypsin digestion

Trypsin digest method was used for the isolation of retinal vessels 49. In brief, the eyeballs were enucleated and fixed in 10% neutral formaldehyde solution for 24 h at 4 ℃. Then, the retinas were dissected, shaken in water at room temperature overnight, and digested with 3% trypsin (1:250, BD Difco, USA, Cat. 215250) in 0.1 M Tris buffer (pH 7.8) at 37 ℃. When the retinas begun disintegration, they were gently washed to free retinal vessels. The free vessels were mounted on adhesive glass slides for dry and stained with periodic acid-schiff and hematoxylin (PAS-hematoxylin). The number of acellular capillaries was qualified from 10 to 20 random fields.

### Cell culture and treatment

Human umbilical vein endothelial cells (HUVECs) and human retinal microvascular endothelial cells (HRVECs) were obtained from American type culture collection (ATCC) and Cell Systems Corporation, respectively. They were cultured in endothelial cell medium (ECM, ScienCell, USA, Cat.1001) containing 10% fetal bovine serum (FBS, ScienCell, USA, Cat. 0025) and 1% penicillin-streptomycin (Gibco, USA) with endothelial cell growth supplements (ECGS, ScienCell, USA, Cat. 1052) at 37 ℃ with 5% CO_2_. To mimic hypoxic condition *in vitro*, these cells were incubated with CoCl_2_ (300 μmol/L) for 24 h. To mimic hyperglycemic condition *in vitro*, they were exposed to high glucose (30 mM D-glucose) for 24 h. Aflibercept (40 μg/ml) was used as the anti-angiogenic control to estimate the angiogenic role of tsRNA-1599.

### tsRNA transfection

tsRNA-1599 mimic, inhibitor, and the corresponding negative controls were designed and synthesized by RiboBio (Guangzhou, China). When endothelial cells grew to 70%-80% confluence, Lipofectamine RNAiMAX Transfection Reagent (Invitrogen, USA, Cat. 13778075) was used to introduce the mimic and inhibitor into HUVECs or HRVECs according to the manufacturer's protocol. The final concentration of mimic and inhibitor in each well was 30 nM. The sequences of tsRNA-1599 mimic, inhibitor, and the negative controls were listed in Table S2.

### Determinations of extracellular acid rate (ECAR)

ECAR was detected using the Seahorse XFe96 Extracellular Flux Analyzer (Seahorse Bioscience, MA, USA). Briefly, HUVECs (10,000 cells per well) were seeded into a Seahorse XFe-96 well culture plate containing 100 μL of ECM supplemented with 10% FBS following the required treatment. Then, the cells were incubated with pyruvate‑free glycolytic assay medium for 1 h prior to first injection of a saturated concentration of glucose. The second injection was oligomycin, which may divert energy production to glycolysis by restricting mitochondrial ATP production. The final injection was 2‑deoxy‑glucose, which was a glucose analog that inhibited glycolysis through competitive binding to glucose hexokinase. For ECAR assay, glucose (10 mM), oligomycin (2 μM), and 2-deoxyglucose (2-DG, 100 mM) were injected into each well.

### Biotin RNA pull down and mass spectrometry

RNA pull down assay was performed as previously described 50. In brief, ECs were lysed on ice for 1 h in the lysis buffer with the protease inhibitor cocktail (1 X) and RNase inhibitor (1 U/μL), followed by centrifugation at 12, 000 *g* at 4 °C for 10 min. Then, the supernatants were transferred to a new tube and incubated with tsRNA-1599 mimic/scramble tsRNA biotin-labeled probes (RiboBio, Guangzhou, China) overnight at 4°C. Subsequently, the streptavidin beads (Smart-Lifesciences, Cat. SM017001) were washed with the diluted buffer for 3 times. Then, the supernatant was rotated with streptavidin beads at room temperature for 2 h. Finally, the beads were washed with the wash buffer for 3 times and suspended with the diluted buffer followed by immunoblots and mass spectrometry.

### RNA immunoprecipitation assay

RIP assay was performed as previously reported 51. Briefly, 2 × 10^7^ of ECs were lysed with RIP lysis buffer and the lysates were incubated with the magnetic beads conjugated to 5 μg of anti-YBX1 (Abcam, ab76149) or anti-IgG (negative controls) overnight at 4 °C. The immunoprecipitated RNAs were eluted, purified, and dissolved in RNase-free water, which were subjected for qRT-PCR analysis of tsRNA-1599 expression.

### Statistical analysis

All results were expressed as mean ± standard deviation (SD) from at least three independent assays. For two-group comparison, the statistical significance was determined by Student's *t*-test or Mann-Whitney *U* test. For multiple comparison, the statistical significance was determined by one-way ANOVA or Kruskal-Wallis test followed by Bonferroni's post-hoc test depending on data distribution and variance. *P <* 0.05 was considered to be statistically significant. GraphPad Prism version 9.1.0 was used for statistical analysis.

## Supplementary Material

Supplementary methods, figures and tables.

## Abbreviations

AH: Aqueous humor
CNV: Choroidal neovascularization
CoCl2: Cobalt chloride
DR: Diabetic retinopathy
ECAR: Extracellular acidification rate
ENO2: Enolase 2
FISH: Fluorescent in situ hybridization kit
HK2: Hexokinase 2
HRVECs: Human retinal microvascular endothelial cells
HUVECs: Human umbilical vein endothelial cells
nAMD: Neovascular age-related macular degeneration
OIR: Oxygen-induced retinopathy model
PAS: Periodic Acid-Schiff stain
PFKFB3: fructose‑2,6‑biphosphatase 3
PKM2: Pyruvate kinase 2
tsRNAs: tRNA -derived small RNAs
VEGF: Vascular endothelial growth factor
YBX1: Y-Box binding protein 1
