# Supplementary material for: Targeting endothelial glycolytic reprogramming by tsRNA-1599 for ocular anti-angiogenesis therapy
Source: Theranostics. 2024 Jun 1;14(9):3509–25. doi: 10.7150/thno.96946 (PMC11209708; doi:10.7150/thno.96946)
Supplement: Supplementary file 1 — Supplementary methods, figures and tables. [file thnov14p3509s1.pdf]

Supplemental data

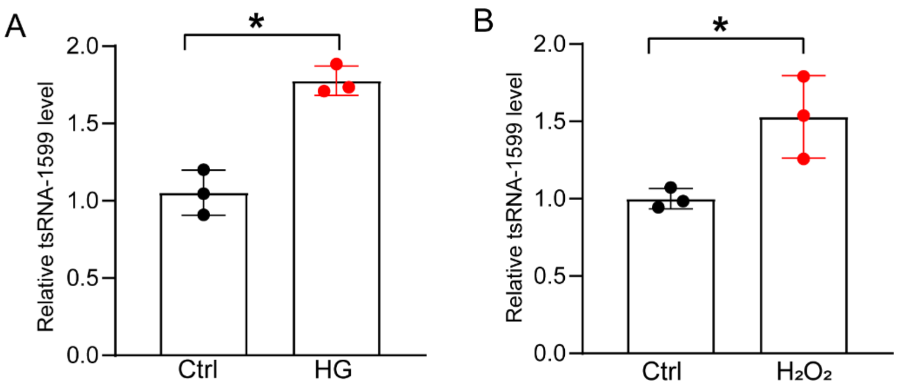

Supplementary Figure 1. tsRNA-1599 expression is up-regulated following high glucose stress and oxidative stress

(A and B) HUVECs were exposed to high glucose (30 mM), H_2O_2 (300 μM) or left untreated (Ctrl) for 24 h. The levels of tsRNA-1599 expression were detected by qRT-PCR assays (n = 3, *P < 0.05 vs. Ctrl, Student t test).

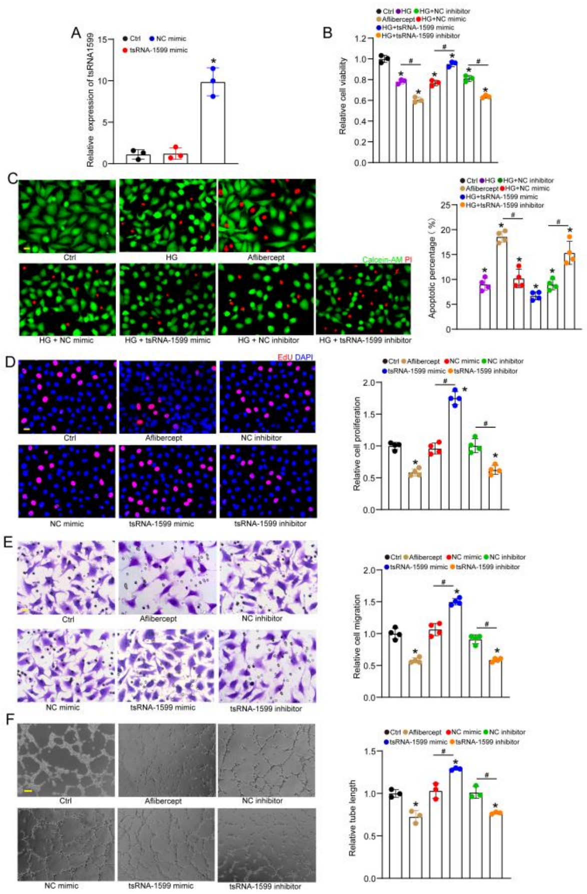

Supplementary Figure 2. tsRNA-1599 regulate angiogenic effects in HRVECs

(A) HRVECs were transfected with negative control (NC) mimic (30 nM), tsRNA-1599 mimic (30 nM), or left untreated (Ctrl) for 24 h. tsRNA-1599 expression was detected by qRT-PCRs (**P* < 0.05 vs. Ctrl group, n = 3). (B and C) HRVECs were transfected with NC mimic (30 nM), tsRNA-1599 mimic (30 nM), NC inhibitor (30 nM), tsRNA-1599 inhibitor (30 nM), treated with aflibercept (40 μg/mL), or left untreated (Ctrl) for 24 h, and then treated with or without high glucose for 24 h. The viability of HRVECs was determined by CCK-8 assays (B, n = 3). Calcein-AM/PI assay was conducted to detect cell apoptosis (C, n = 4, Scale bar, 20 μm). (D - F) HRVECs were transfected with NC mimic (30 nM), tsRNA-1599 mimic (30 nM), NC inhibitor (30 nM), tsRNA-1599 inhibitor (30 nM), treated with aflibercept (40 μg/mL), or left untreated (Ctrl) for 24 h. The proliferation ability was determined by EdU assays (D, n = 4, Scale bar, 20 μm). Cell migration and quantitative analysis was conducted by transwell assays (E, n = 4, Scale bar, 20 μm). Tube formation assays and quantitative analysis were conducted to detect tube formation ability (F, n = 3, Scale bar, 50 μm). **P* < 0.05 vs. Ctrl group; $^{\#}P$ < 0.05 between the marked group; One-way ANOVA followed by Bonferroni's post hoc test.

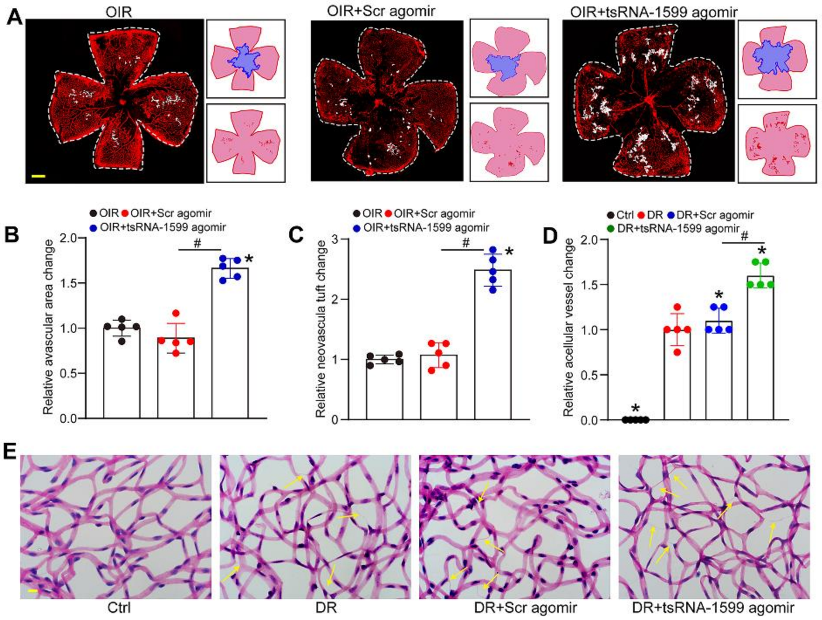

Supplementary Figure 3. tsRNA-1599 agomir contributes to pathological angiogenesis in vivo

(A) IB4 staining of whole-mount retinas from OIR, Scr agomir (20 μM, 1 μL), and tsRNA-1599 agomir-injected OIR mice (20 μM, 1 μL) at P17 (n = 5 mice) with the quantification of avascular areas and NVTs. The white line denotes retinal margin and the white area represents NVTs. In the insets, red line: retinal margin; blue area: avascular area; red area: NVTs. (B and C) Quantification analysis of avascular areas and NVTs, respectively. $*P <$ 0.05 vs. OIR group; One-way ANOVA followed by Bonferroni's post hoc test. (D and E) Retinal trypsin digestion was conducted to detect the number of acellular capillaries in non-DR mice (Ctrl), DR mice, DR mice-injected Scr antagomir (20 μM, 2 μL), tsRNA-1599 antagomir (20 μM, 2 μL), or aflibercept (40 mg/mL, 2 μL). Yellow arrows indicated acellular capillaries (n = 5 mice for each group, Scale bar, 10 μm). $*P < 0.05$ vs. DR group; $^{\#}P < 0.05$ between the marked group; Scr, scramble; NVT, neovascular tuft; DR, diabetic retinopathy; One-way ANOVA followed by Bonferroni's post hoc test.

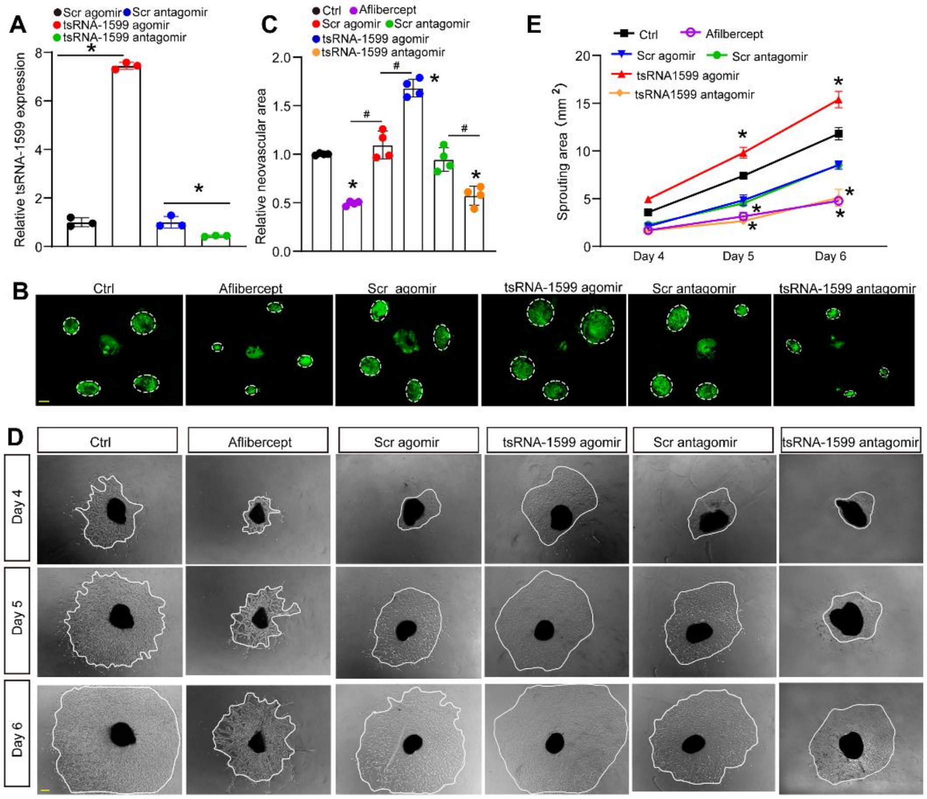

Supplementary Figure 4. tsRNA-1599 silencing plays an anti-angiogenic role in choroidal neovascularization

(A) C57BL/6 mice received injections of scrambled (Scr) agomir (20 μM, 2 μL), tsRNA-1599 agomir (20 μM, 2 μL)， Scr antagomir (20 μM, 2 μL), or tsRNA-1599 antagomir (20 μM, 2 μL) for 14 days. qRT-PCRs were conducted to detect the levels of tsRNA-1599 in the choroid/RPE tissues (n = 3, Student *t* test). (B and C) C57BL/6 mice received intravitreal injections of Scr agomir (20 μM, 2 μL), tsRNA-1599 agomir (20 μM, 2 μL), Scr antagomir (20 μM, 2 μL), tsRNA-1599 antagomir (20 μM, 2 μL), aflibercept (40 mg/mL, 2 μL), or left untreated (Ctrl). At day 14 following laser photocoagulation, the mice were euthanized and the RPE/choroid complexes were dissected and flat-mounted for IB4 labeling. The lesions were highlighted by white circles. Four spots per eye were averaged (n = 4, Scale bar, 200 μm). $^{*}P < 0.05$ vs. Ctrl group; $^{\#}P < 0.05$ between the marked group; One-way ANOVA followed by Bonferroni's post hoc test. (D and E) C57BL/6 mice received intravitreal injections of Scr agomir (20 μM, 2 μL), tsRNA-1599 agomir (20 μM, 2 μL), Scr antagomir (20 μM, 2 μL), tsRNA-1599 antagomir (20 μM, 2 μL), aflibercept (40 mg/mL, 2 μL), or left untreated (Ctrl). At day 14, RPE/choroid complexes were dissected, cut into 1 mm × 1 mm pieces, and seeded into 24-well plates. The sprouting potency of choroidal explants were photographed on day 4, day 5, and day 6 (n = 4, Scale bar, 500 μm). $^{*}P < 0.05$ vs. Ctrl group; One-way ANOVA followed by Bonferroni's post hoc test.

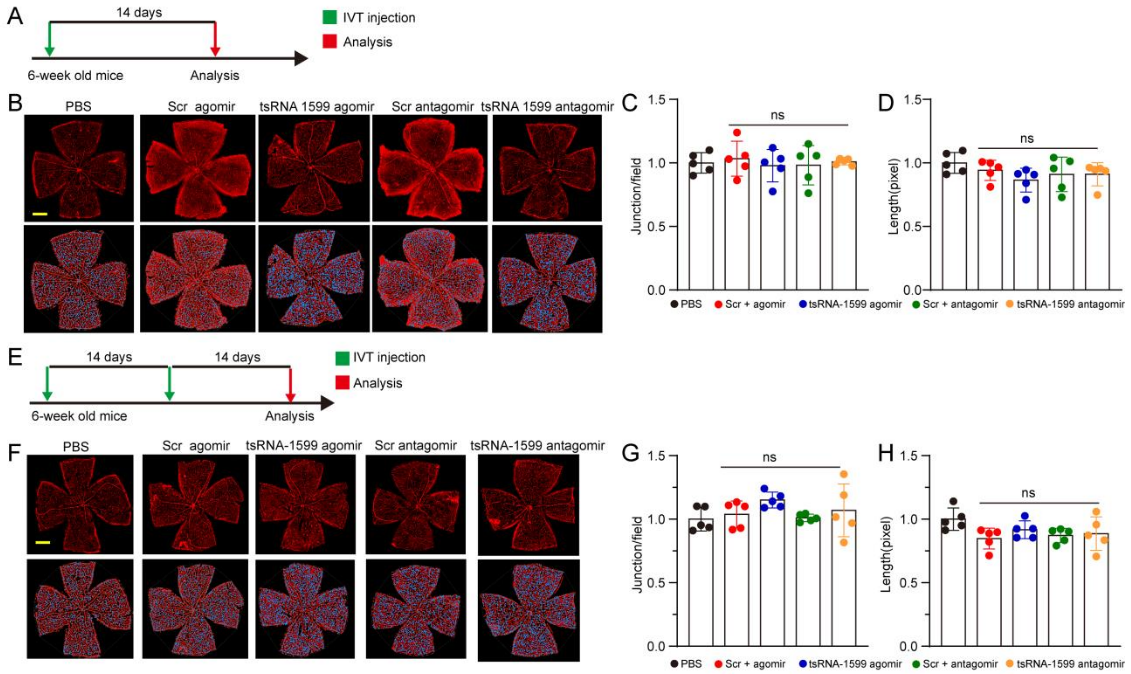

Supplementary Figure 5. Injection of tsRNA-1599 agomir and antagomir does not destroy the existing vessels

(A) Diagram illustrating the experimental procedure for assessing the effects of altered tsRNA-1599 levels on the existing vessels for 14 days. (B - D) IB4 staining of retinal flat-mounts injected with scramble (Scr) agomir (20 μM, 2 μL), tsRNA-1599 agomir (20 μM, 2 μL), Scr antagomir (20 μM, 2 μL), tsRNA-1599 antagomir (20 μM, 2 μL), or left untreated (PBS) for 14 days (B, Scale bar, 500 μm). Quantification of vascular junctions and lengths were shown (C and D, n = 5). (E) The diagram illustrating experimental procedure for assessing the effects of altered tsRNA-1599 levels on the existing vessels for 28 days. (F-H) IB4 staining of retinal flat-mounts injected with Scr agomir (20 μM, 2 μL), tsRNA-1599 agomir (20 μM, 2 μL), Scr antagomir (20 μM, 2 μL), tsRNA-1599 antagomir (20 μM, 2 μL), or left untreated (PBS) for 28 days (F, Scale bar, 500 μm). Quantification of vascular junctions and lengths were shown (G and H, n = 5). “ns ” represents no statistical significance; One-way ANOVA followed by Bonferroni’s post hoc test.

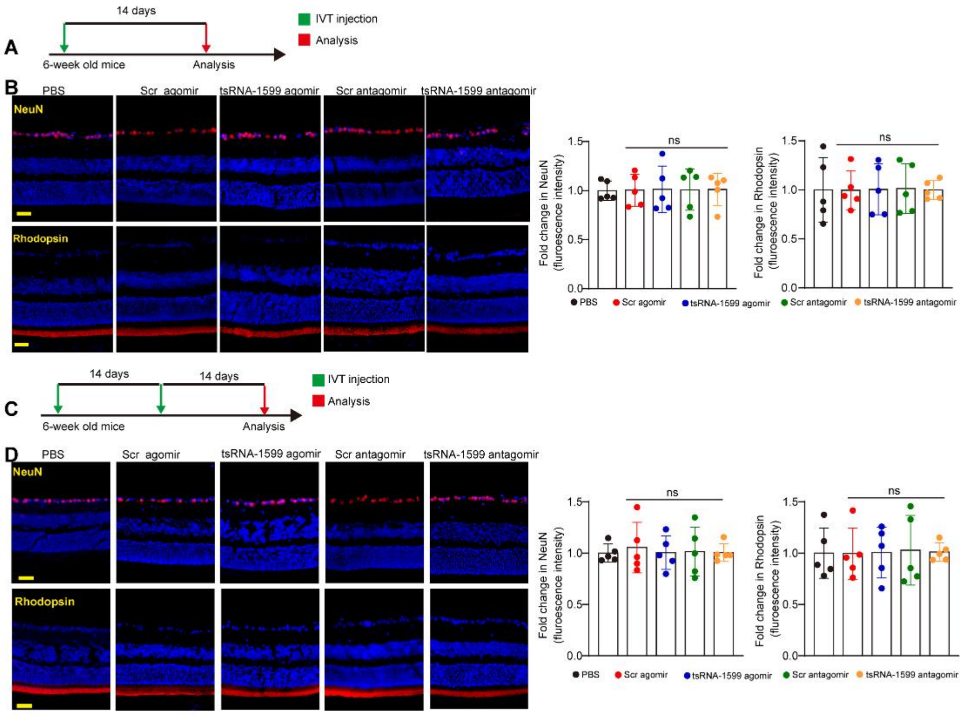

Supplementary Figure 6. tsRNA-1599 injection does not cause retinal neurodegeneration

(A) Diagram illustrating the experimental procedure for assessing the effects of altered tsRNA-1599 levels on retinal neuronal toxicity for 14 days. (B) Immunofluorescence staining of retinas injected with Scr agomir (20 µM, 2 µL), tsRNA-1599 agomir (20 µM, 2 µL), Scr antagomir (20 µM, 2 µL), tsRNA-1599 antagomir (20 µM, 2 µL), or PBS for 14 days with NeuN and Rhodopsin (Scale bar, 50 µm). Quantification results and representative images of NeuN and Rhodopsin staining were shown (n = 5). (C) Diagram illustrating the experimental procedure for assessing the effects of altered tsRNA-1599 levels on retinal neuronal toxicity for 28 days. (D) Immunofluorescence staining of retinas injected with Scr agomir (20 µM, 2 µL), tsRNA-1599 agomir (20 µM, 2 µL), Scr antagomir (20 µM, 2 µL), tsRNA-1599 antagomir (20 µM, 2 µL), or PBS for 28 days with NeuN and Rhodopsin (Scale bar, 50 µm). Quantification results and representative images of NeuN and Rhodopsin staining were shown (n = 5). "ns " represents no statistical significance; One-way ANOVA followed by Bonferroni test.

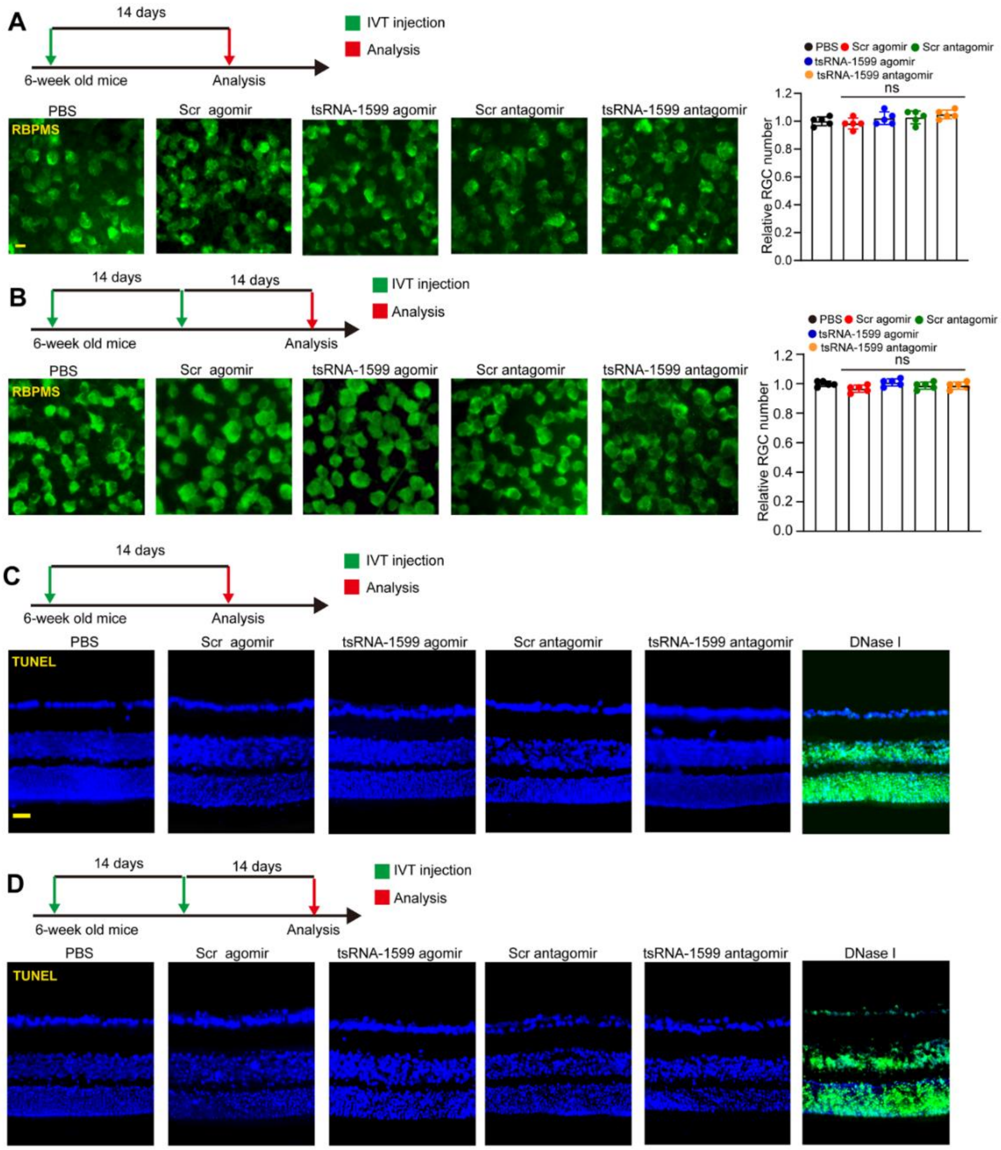

Supplementary Figure 7. tsRNA-1599 injection does not cause RGC injury and retinal apoptosis

(A and B) The retinas were injected with Scr agomir (20 μM, 2 μL), tsRNA-1599 agomir (20 μM, 2 μL), Scr antagomir (20 μM, 2 μL), tsRNA-1599 antagomir (20 μM, 2 μL), or PBS for 14 days or 28 days with RBPMS (Scale bar, 20 μm). Representative images of flat-mounted retinas stained with RBPMS. Displayed images were taken half of retinal radius from the center of retina. Quantification results and representative images of RBPMS staining are depicted. (C and D) TUNEL staining of retinas injected with Scr agomir (20 μM, 2 μL), tsRNA-1599 agomir (20 μM, 2 μL), Scr antagomir (20 μM, 2 μL), tsRNA-1599 antagomir (20 μM, 2 μL), or PBS for 14 days or 28 days (Scale bar, 50 μm). n = 5; "ns" represents no statistical significance; One-way ANOVA followed by Bonferroni test.

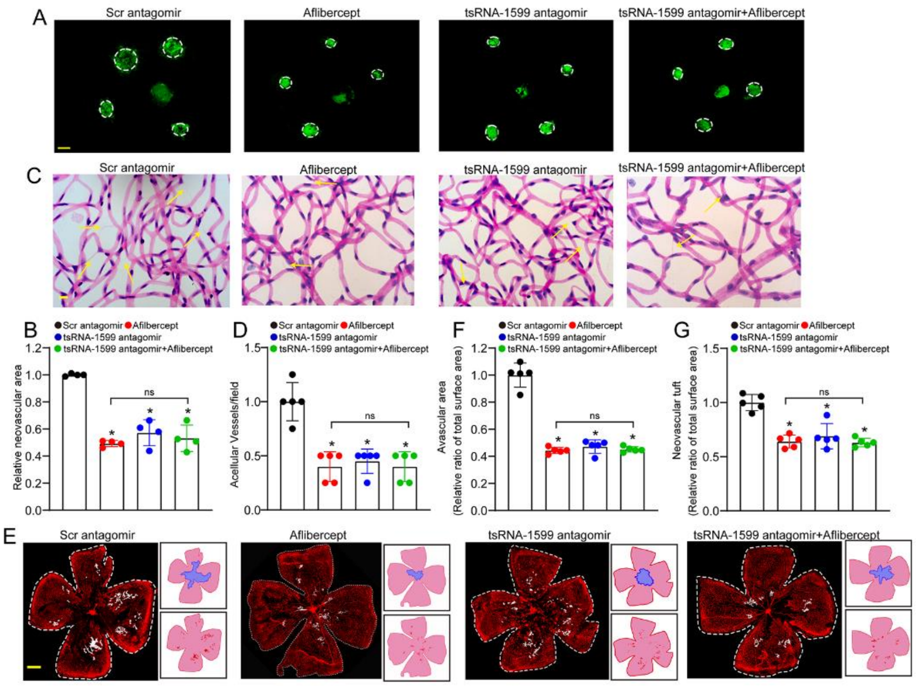

Supplementary Figure 8. The efficacy of combined use of tsRNA-1599 and aflibercept (A and B) C57BL/6 mice received an intravitreal injection of Scr antagomir (20 µM, 2 µL), tsRNA-1599 antagomir (20 µM, 2 µL), tsRNA-1599 antagomir plus aflibercept or aflibercept (40 mg/mL, 2 µL). At day 14 after laser photocoagulation, the RPE/choroid complexes were dissected and flat-mounted for IB4 labeling. The lesions were highlighted by the white circles. Four spots per eye were averaged. Scale bar, 200 µm. (C and D) Retinal trypsin digestion was conducted to detect retinal acellular capillaries in DR mice-injected Scr atagomir (20 µM, 2 µL), DR mice-injected tsRNA-1599 antagomir (20 µM, 2 µL), DR mice-injected tsRNA-1599 antagomir plus aflibercept or afliberceptor (40 mg/mL, 2 µL). Yellow arrows indicated acellular capillaries. Scale bar, 10 µm. (E) IB4 staining of whole-mount retinas from OIR mice-injected Scr atagomir (20 µM, 1 µL), OIR mice-injected tsRNA-1599 antagomir (20 µM, 1 µL), OIR mice-injected tsRNA-1599 antagomir plus aflibercept or aflibercept (40 mg/mL, 1 µL) at P17 (Scale bar, 500 µm) with the quantification of avascular areas and neovascular tuft (NVT) areas. The white line denotes retinal margin and the white area represents NVTs. In the insets, red line: retinal margin; blue area: avascular area; red area: NVTs. (F and G) Quantitative analysis of avascular area and neovascular tuft (NVT) area. *$P < 0.05$ vs. Scr antagomir group; "ns" represents no statistical significance; One-way ANOVA followed by Bonferroni's post hoc test.

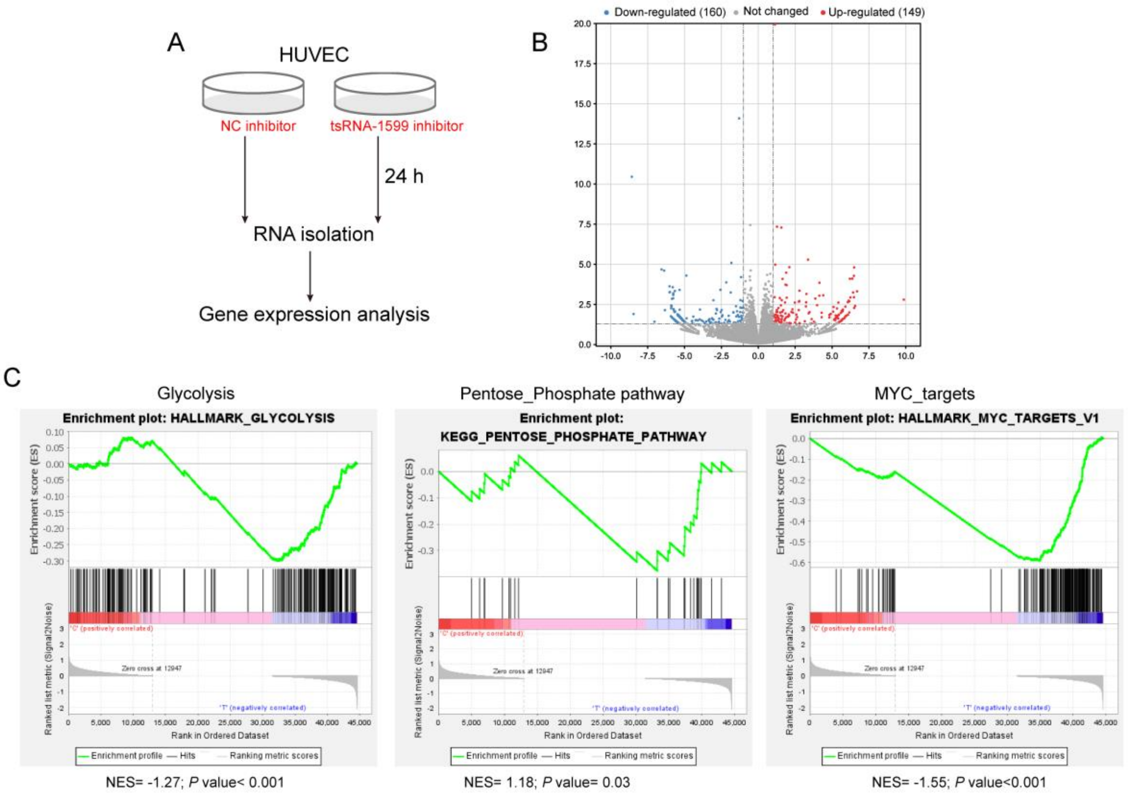

Supplementary Figure 9. Transcriptional analysis of tsRNA-1599 inhibitor stimulation in HUVECs

(A) HUVECs were transfected with negative control (NC) inhibitor (30 nM) or tsRNA-1599 inhibitor (30 nM) for 24 h. RNA-seq analysis was conducted to identify differentially expressed genes following tsRNA-1599 inhibition (n = 3). (B) Volcano plot displaying the differentially expressed genes between NC inhibitor-transfected group and tsRNA-1599 inhibitor-transfected group. (C) GSEA pathway analysis of NC inhibitor-transfected vs. tsRNA-1599 inhibitor-transfected HUVECs for glycolysis, pentose_phosphate pathway, and MYC_target signaling pathway.

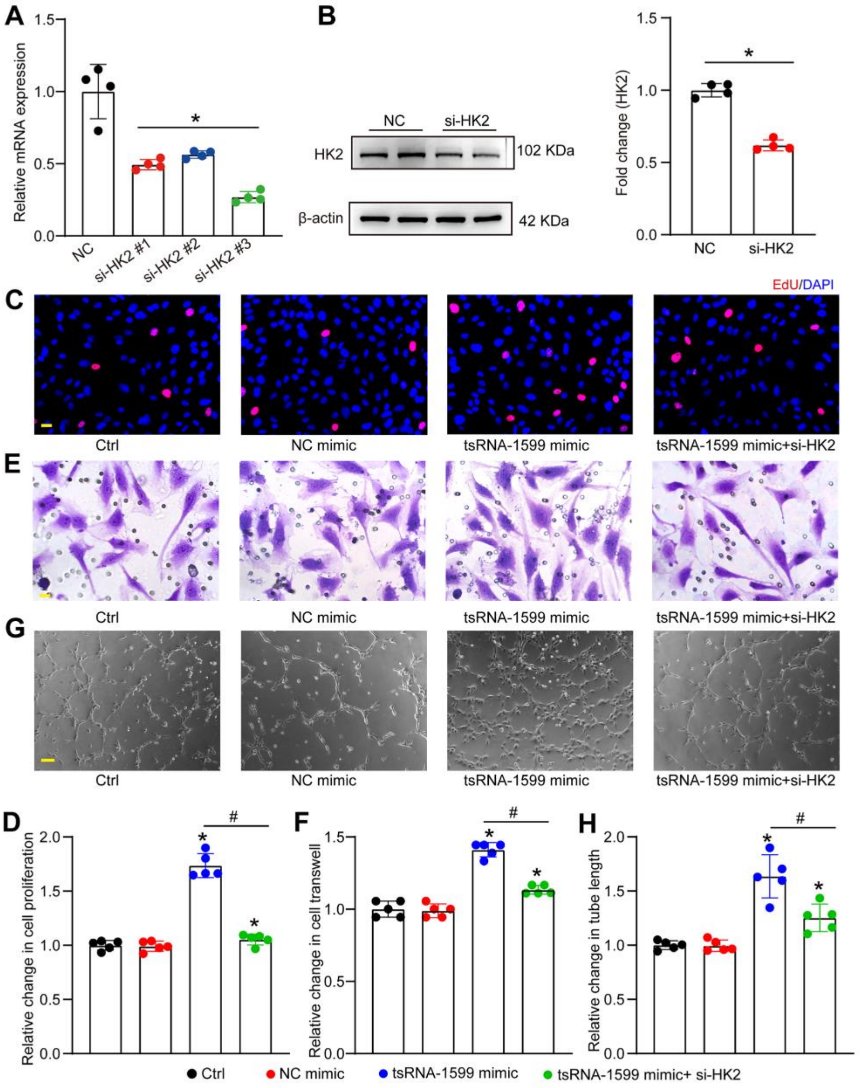

Supplementary Figure 10. HK2 was identified as a downstream gene of tsRNA-1599

(A and B) HUVECs were transfected with negative control (NC) siRNA (30 nM) or HK2 siRNA 1-3 (30 nM) for 24 h. qPCR assays (A, n = 4, **P* < 0.05 vs. NC group, One-way ANOVA followed by Bonferroni's post hoc test) and western blots were conducted to detect the expression of YBX1 (B, n = 4, **P* < 0.05 vs. NC group, Student *t* test). (C - H) HUVECs were transfected with NC mimic (30 nM), tsRNA-1599 mimic (30 nM), tsRNA-1599 mimic (30 nM) plus HK2 siRNA (30 nM), or left untreated (Ctrl) for 24 h, and then treated with $CoCl_2$ (300 μmol/L) for 24 h. The proliferation ability of HUVECs was determined by EdU assays (C and D, n = 5, Scale bar, 20 μm). Cell migration and quantitative analysis was conducted by transwell assays (E and F, n = 5, Scale bar, 20 μm). Tube formation assays and quantitative analysis were conducted to detect tube formation ability (G-H, n = 5, Scale bar, 50 μm). **P* < 0.05 vs. Ctrl group; $^{\#}P$ < 0.05 between the marked group; One-way ANOVA followed by Bonferroni's post hoc test.

Table S1: Top 5 proteins interacting with tsRNA-1599 identified by mass spectrometry

No.	Entry name	MW (kDa)	Unique peptides	Score
1	NONO	54	13	87.44
2	YBX1	35	4	36.28
3	YBX3	40	3	24.38
4	LYAR	43	3	9.27
5	PCBP1	37	4	8.84

Table S2: RNA oligonucleotide sequences

Gene	Sequence (5'-3')	Sequence (3'-5')
tsRNA-1599 mimic	UAGGUCGCUGGUUCGUUUCC	/
tsRNA-1599 inhibitor	GGAAACGAACCAGCGACCUA	/
NC mimic	CGGGUUAUGUCGCCUUCUGU	/
NC inhibitor	CGGGUUAUGUCGCCUUCUGU	/
tsRNA-1599 antagomir	GGAAACGAACCAGCGACCUA	/
tsRNA-1599 agomir	UAGGUCGCUGGUUCGUUUCC	AAACGAACCAGCGACCUAUU
Scramble antagomir	CAGUACUUUUGUGUAGUACAA	/
Scramble agomir	UUCUCCGAACGUGUCACGUTT	ACGUGACACGUUCGGAGAATT

Table S3: Primer sequences used for qPCR assays for tsRNA expression

Genes	Forward (5'-3')
tsRNA-1599	TAGGTCGCTGGTTCGTTTC
tsRNA-1632	TTCGTTTCCCGGCCAA
tsRNA-1536	CATATTGTCTAGCGGTTAGGATTC
tsRNA-1636	GGGCATTGGTAGTTCAATGGTAG
tsRNA-1598	CGGTCTAAGGCTCTGCGTTC
tsRNA-117	GCGGAAGCGTACTGGGC
tsRNA-124	GGGCTCTGTGACGCAATG

Table S4: Primers used for qPCR assays for mRNA expression

Genes	Forward (5'-3')	Reverse (3'-5')
YBX1	TGCAGGGAGAAGTGATGGAG	TTAGGGTTTTCTGGGCGTCT
HK2	TAGGGCTTTGAGAGCACCTGT	CCACACCACTGTCACTTTG
ENO2	AGGTGCAGAGGTCTACCATAC	AGCTCCAAGGCTTCACTGTTC
PKM2	ATGTCGAAGCCCCATAGTGAA	TGGGTGGTGAATCAATGTCCA
PFKFB3	CGCCTACCTCAACGTGGTG	ACCTCCAGAACGAAGGTCCTC

Table S5: Primers used for ChIP qPCR assay

Genes	Forward (5'-3')	Reverse (3'-5')
HK2 site 1	CTTTCCGTCCCAGCCTTTAG	TTAGGGTTTTCTGGGCGTCT
HK2 site 2	CGTGTGCTGGTCCAGAG	TAAAGGCTGGGACGGAAAG

Supplemental materials and methods

Clinical sample collection

The clinical samples were obtained from 2021 to 2023 at the Affiliated Eye Hospital of Nanjing Medical University, in accordance with the Declaration of Helsinki. Informed consents were obtained from all patients before inclusion. Aqueous humor (AH) was collected prior to intravitreal anti-VEGF therapy (for nAMD and PDR group) or prior to phacoemulsification surgery (for control group).

Intravitreal injection

C57BL/6J mice were anesthetized via intraperitoneal injection of ketamine (80 mg/kg) and xylazine (10 mg/kg). The pupils were dilated with 1% tropicamide eye drops (0.5%; Alcon, USA). Under an operating microscope (66 Vision Tech, China), about 2 μl of tsRNA-1599 agomir/antagomir, negative control agomir/antagomir, or PBS buffer was injected into vitreous body using a 33-guage needle attached to a Hamilton micro syringe through a vitrectomy port (2 mm posterior to the limbus). Supplementary injections were conducted if needed.

Immunofluorescent staining

The eyeballs were placed in the Fekete's solution at 4 °C for 2 h. The cornea, lens, and vitreous humor was removed. The remaining tissues were fixed in 4% paraformaldehyde (PFA; BL539A, Biosharp Biotechnology, China) overnight at 4°C. Then, they were dehydrated in 30% sucrose solution, embedded in OCT compound, and stored at -80 °C until sectioning. The frozen samples were sectioned to 5-μm thickness on a

cryostat (Thermo Scientific, USA) and placed on the adhesion microscope slides (Citotestt, China). The frozen sections were washed with PBS for 3 times, permeabilized, and blocked with 5% bovine serum albumin (BSA) and 1% Triton X-100 for 1 h at 37 ℃. Then, the sectioned tissues were stained with the primary antibodies, including rabbit anti-NeuN (1:200, Abcam, ab177487), rabbit anti-rhodopsin (1:200, Abcam, ab221664) and rabbit anti-RBPMS (1:200, HUABIO, ER1901-43) overnight at 4 ℃. After washing with PBST for 3 times, the sections were incubated the secondary antibodies: goat anti-rabbit IgG (H+L) (Invitrogen, A11012) or goat anti-mouse IgG (H+L) (Invitrogen, A11005) at room temperature for 2 h. The nuclei were stained with DAPI for 5 min. The images were photographed by a fluorescence microscope (Olympus IX70, Japan) and analyzed by Image J software.

TUNEL assay

The apoptosis of ocular tissues was assessed using TUNEL assays with the *In Situ* Cell Detection Kit (C1086, Beyotime, China). In brief, paraffin-embedded tissue sections were deparaffinized with xylene and rehydrated through a series of alcohol gradients followed by distilled water. The sections were then treated with permeabilization solution (containing 20 μg/ml proteinase K in 10 mM Tris/HCl) at room temperature for 15 min and rinsed with distilled water. Subsequently, the sections were incubated with the TUNEL reaction mixture for 1 h at 37°C. The nuclei were counterstained with DAPI solution for 5 min. Finally, the sections were visualized under a fluorescence microscope (Olympus, Tokyo, Japan).

Quantification of avascular area and neovascular tuft area in OIR retinas

Retinal images were imported into Adobe Photoshop 2021. The entire retina, avascular area, and retinal neovascularization (NV) area were selected using the polygonal lasso tool, quick selection tool, and magic wand tool, respectively. Finally, pixel measurements were conducted and recorded for the subsequent analysis.

Quantification of RGCs stained with RBPMS

To assess RGC number, the images of whole-mounted retinas were captured using a fluorescence microscope at 40 × magnification. Six sample areas were chosen from each flat-mounted retina stained with anti-RBPMS: two from the central, mid-peripheral, and peripheral retina regions (approximately 1/6, 3/6, and 5/6 of the retinal radius from the optic nerve head, respectively). The number of RBPMS-positive cells was counted in each area and cell count was derived from the average across the three retinal regions.

Quantification of vascular length and branch point number

Retinal images were imported into AngioTool 64.0 (https://ccrod.cancer.gov/confluence/display/ROB2/Home). The identified vessels are demarcated with an outline on the displayed image. Then, the parameter of "vessel diameter and intensity" was set as 8-20. Meantime, the image dynamically updated its shape in response to the adjustments done using the controls included in the analysis tab. Once the outline overlay matched the vessels in the displayed image, the analysis was conducted. Finally, AngioTool was used to calculate vascular length and branch points.

Cell Counting Kit-8 assay

Cell viability was evaluated by Cell Counting Kit-8 (CCK-8, Beyotime, C0071S) assays according to manufacturer's instruction. ECs were re-suspended and seeded in a 96-well plate. Following the required treatment, 100 μl of CCK-8 solution diluted by ECM (1:10) was added to each well of 96-well plate. These cells were incubated for 2 h. Finally, the absorbance at 450 nm was determined by a microplate reader (FilterMax F5, Molecular Devices).

Cell proliferation assay

Cell proliferation was evaluated by an EdU kit (Beyotime, China, C0071S). Briefly, ECs were cultured into 24-well plates. EdU staining reagent was added to each well and incubated at 37 °C for 2 h. Then, these cells were fixed with 4% PFA and permeabilized with 0.3% Triton X-100 for 15 min. After washing, these cells were stained with the click reaction solution for 30 min to label the proliferating cells. Cell nuclei was stained with 4′, 6-diamidino-2-phenylindole (DAPI, Beyotime, C1002) for 10 min at room temperature. The stained cells were observed by a fluorescence microscope (Olympus IX70, Japan).

Calcein-AM/propidium iodide (PI) double staining

Cell apoptosis was determined by Calcein-AM/propidium iodide (PI) double staining (Beyotime, C2015S). Briefly, ECs were seeded into 24-well plates followed the required treatment. An equal volume of PI (10 μmol/L) and Calcein (10 μmol/L) was added to each well of plate and incubated with the cells for 20 min at room temperature. The images were observed by a fluorescence microscope (Olympus IX70, Japan).

Transwell assay

Cell migration was determined by Transwell assays. ECs were seeded into the transwell chamber (8 μm pore size; Costar, USA) in 24-well plates. After the required treatment, these cells were re-suspended in serum-free ECM medium and 100 μl of cell suspension (3.5×10^4 cells) was added to the upper chamber. 500 μl of complete medium was added to the lower chamber. These cells were cultured for 20 h at 37 °C and the non-migrated cells in the upper chamber were removed. The cells attached the lower surface were fixed with methanol for 15 min and stained with the crystal violet (C805211, Macklin) for 10 min. The images were observed by a phase-contrast microscope (Olympus, Tokyo, Japan).

Tube formation assay

The 96-well plates were pre-cooled and pre-coated with Matrigel (Corning, NY, USA, CAT#356230) and returned to CO_2 incubator for gel solidification at 37 °C for 30 min. After the required treatment, ECs were digested with trypsin and seeded onto Matrigel (3×10^5 cells per well) in ECM medium containing 10% FBS. After 8 h-culture at 37 °C in 5% CO_2, the total tube lengths in each well were measured by Image J software.

Quantitative reverse transcription PCR assay

Total RNAs were extracted from the cell lysates or the retinas with an RNA isoplus kit (Takara, Kyoto, Japan). The extracted RNAs were reversely transcribed into cDNAs using a microRNA Reverse Transcription Kit (EZB-miRT4, EZBioscience, China) with the specific stem-loop RT-primer according to the manufacturer's instruction. PCR products were amplified using the EZ-probe qPCR Master Mix (EZB-miProbe-R2, EZBioscience,

China) on an Applied Biosystems Step One Real-time system (Applied Biosystems). For other genes, RNA samples were reversely transcribed into cDNAs using the HiScript III RT SuperMix (R323, Vazyme Biotech Co.,Ltd, China). qPCR assays were conducted using the ChamQ SYBR qPCR Master Mix (Q321, Vazyme Biotech Co.,Ltd, China). Relative tsRNA expression was normalized to U6 and other genes were normalized β-actin. Each reaction was performed at least in triplicate and calculated by the $2^{-\Delta\Delta Ct}$ method. The primer sequences were shown in Table S3 and S4.

Western blot

Total proteins were extracted from ECs using RIPA lysis buffer (Beyotime Biotech, Shanghai, China) supplemented with protease inhibitor cocktail and phosphatase inhibitors. Protein concentration was determined using the bicinchoninic acid (BCA) protein assay kit (Beyotime Biotechnology, Shanghai, China). Equal amounts of proteins (10-15 μg) were separated by SDS-PAGE and transferred onto polyvinylidene difluoride (PVDF) membranes (EMD Millipore). The membranes were blocked with 5% defatted milk in Tris-buffered saline with 0.05% Tween 20 (TBST). Subsequently, the membranes were incubated with the primary antibodies: VEGFR2 (1:1000, Cell Signaling Technology, CAT#2479), pVEGFR2 (1:1000, Cell Signaling Technology, CAT#2478), Akt (1:1000, Cell Signaling Technology, CAT#4685), p-Akt (Cell Signaling Technology, CAT#4060,1:1000), p38 (1:1000, Cell Signaling Technology, CAT#8690), p-p38(1:1000, Cell Signaling Technology, CAT#4511), ERK (1:1000, Cell Signaling Technology, CAT#4695), p-ERK (1:1000, Cell Signaling Technology, CAT#9101), YBX1 (1:1000,

abcam, ab76149), HK2 (1:1000, abcam, ab209847), PKM2 (1:1000, proteintech, 15822-1-AP), PFKFB3 (1:1000, abcam, ab181861), or β-actin (1:1000, ZENBIO, CAT#38062) overnight at 4 ℃. Then, the membranes were incubated with HRP-labeled Goat Anti-Mouse IgG (H+L) (1:1000, Beyotime, A0216, China) or HRP-labeled Goat Anti-Rabbit IgG (H+L) (1:1000, Beyotime, A0208, China) for 3 h at room temperature. Protein bands were visualized using an enhanced chemiluminescence detection system (Tennon5200, Shanghai, China).

Subcellular fractionation assay

The Cytoplasmic & Nuclear RNA Purification Kit (NGB-21000, Norgen Biotek) was used to extract nucleus and cytoplasmic RNAs following the manufacturer's instructions. Subsequently, qPCR assays were conducted to assess the levels of tsRNA-1599 in the nucleus and cytoplasmic fractions. β-actin served as the cytoplasmic endogenous control, while U6 was used as the nucleus endogenous control.

Fluorescence in situ hybridization assay (FISH)

FISH assays were carried out to detect the subcellular distribution of tsRNA-1599 in ECs. Briefly, ECs were seeded in 24-well plate and fixed with 4% PFA. Then, they were treated with 0.5% Triton X-100 followed by pre-hybridization. Overnight hybridization was performed with 20 μM probe. RNA FISH kit was purchased from RiboBio (Guangzhou, China). FISH experiment was conducted according to the manufacturer's instruction. Cy3-labeled 18S rRNA, U6, and tsRNA-1599 probes were synthesized by RiboBio (Guangzhou, China). The nuclei were labeled with DAPI reagent. Finally, the

cells were visualized by a fluorescence microscope (Olympus, Tokyo, Japan).

Detection of NAD^+/NADH level

The levels of NAD^+/NADH were assessed using a NAD^+/NADH assay kit with WST-8 (Beyotime, China, CAT#S0175). Following the required treatment, ECs were lysed with 200 µl of lysis buffer. Subsequently, 20 µl of cell lysates was added to a 96-well plate to measure NAD^+/NADH levels. Cell suspension was incubated at 60°C for 30 min, followed by the addition of 90 µl of alcohol dehydrogenase and further incubation at 37°C for 10 min. Subsequently, 10 µl of chromogenic solution was added to the plate and incubated at 37°C for 30 min. Finally, the absorbance at 450 nm was measured using a plate reader (FilterMax F5, Molecular Devices).

tsRNA sequencing

Total RNAs were extracted from the RPE-choroid-sclera complex of laser-induced CNV mice and non-CNV control mice using TRIzol (Invitrogen, CA, USA) reagent following the manufacturer's instruction. The concentration and quantity of each RNA sample were determined by a NanoDrop ND-2000 instrument (Thermo Fisher Scientific, DE, USA). Approximately 1 µg of total RNAs from each sample was used to construct small RNA libraries using a small RNA sample preparation kit (Illumina, San Diego, CA, United States). Subsequently, RNAs were reversely transcribed into cDNAs, followed by PCRs to amplify the libraries. Size selection was performed using polyacrylamide gel electrophoresis. Finally, the amplified cDNAs were purified and library quality was assessed using the Agilent 2,100 Bioanalyzer. All libraries were sequenced on an Illumina

sequencing platform (HiSeqTM 2,500).

RNA sequencing

ECs were transfected with or without tsRNA-1599 inhibitors for 24 h. Transcriptomic analysis was conducted using the Illumina HiSeq XTEN/NovaSeq 6000 sequencer (Thermo Fisher Scientific) following the manufacturer's instructions. RNA-seq transcriptome libraries were prepared using the TruSeqTM RNA sample preparation Kit from Illumina (San Diego, CA) with 1 μg of total RNAs. mRNAs were isolated through polyA selection using oligo (dT) beads and fragmented in a fragmentation buffer. Subsequently, double-stranded cDNA was synthesized using a SuperScript double-stranded cDNA synthesis kit (Invitrogen, CA) with random hexamer primers (Illumina). The synthesized cDNAs underwent end-repair, phosphorylation, and 'A' base addition as per Illumina's library construction protocol. The libraries were size-selected for cDNA fragments of 300 bp on 2% Low Range Ultra Agarose and PCR assays amplified using Phusion DNA polymerase (NEB) for 15 PCR cycles. After the quantification with TBS380, the paired-end RNA-seq libraries were sequenced using the Illumina HiSeq XTEN/NovaSeq 6000 sequencer.

SeqPrep (https://github.com/jstjohn/SeqPrep), Sickle (https://github.com/najoshi/sickle), and HISAT2 (http://ccb.jhu.edu/software/hisat2/index.shtml) were employed for trimming raw paired-end reads, quality control, and alignment to human reference genome. The mapped reads were assembled using StringTie

(https://ccb.jhu.edu/software/stringtie/index.shtml?t=example). To identify differential expression genes (DEGs), the expression level of each transcript was calculated using the transcripts per million reads (TPM) method and determined using the DEseq2 (version 3.11) package. Gene set enrichment analysis (GSEA) was conducted using GSEA software with pre-defined gene sets based on prior biological knowledge.

Detection of the levels of glucose and pyruvate

Following the required treatment, the culture medium of ECs was collected and centrifuged at 12, 000 *g* for 5 min and stored at -80 °C. The level of glucose was detected by mixing culture medium with glucose detection mix. The absorbance was measured at 620 nm wavelength (Beyotime, China, CAT#S0201S). The level of pyruvate was determined using the Pyruvate Colorimetric/Fluorometric Assay Kit (BioVision) according to the manufacturer's instruction.

Chromatin immunoprecipitation (ChIP)

ChIP assays were performed using the ChIP-IT®Express Enzymatic Magnetic Chromatin Immunoprecipitation Kit (Active Motif) according to the manufacturer's instruction. Briefly, 1.5×10^7 of ECs were fixed in 1% formaldehyde for 10 min at 37 °C, washed twice using ice cold PBS, followed by 1 × Glycine Buffer to stop the reaction. Subsequently, these cells were scraped into the tube and lysed with the lysis buffer supplemented with the protease inhibitor cocktail and PMSF. The chromatin DNA was digested by the enzyme at 37 °C for 10 min. After centrifugation, the supernatant was transferred to a new tube and diluted with the ChIP dilution buffer containing the protease

inhibitors. The supernatant fraction was collected and mixed with YBX1 antibody (Proteintech, 20339-1-AP), Protein G magnetic beads, and Protein G magnetic beads cocktail, which were incubated overnight at 4 °C with the rotation. IgG was served as a control. The supernatants contained the unbound and non-specific DNA was removed using the magnetic rack. The magnetic beads were washed using the ChIP buffer. After that, the elution buffer and reverse cross-linking buffer was used to separate the specific DNA. DNA was then treated by proteinase K at 37°C for 1 h followed by incubating with proteinase K stop solution to end the reaction. Finally, the DNA was used for ChIP-qPCR analysis. The ChIP-qPCR primer sequences are given in Table S5.
